# Supplementary material for: CONGA: Copy number variation genotyping in ancient genomes and low-coverage sequencing data
Source: PLoS Comput Biol. 2022 Dec 14;18(12):e1010788. doi: 10.1371/journal.pcbi.1010788 (PMC9873172; doi:10.1371/journal.pcbi.1010788)
Supplement: S1 Text — (PDF) [file pcbi.1010788.s001.pdf]

(Supplementary)
CONGA: Copy Number variation genotyping in ancient genomes and low-coverage sequencing data

Arda Söylev[1,2], Sevim Seda Çokoğlu[2], Dilek Koptekin[2,3], Can Alkan[4], Mehmet Somel[2]

[1] Department of Computer Engineering, Konya Food and Agriculture University, Konya, Turkey
[2] Institute for Medical Biometry and Bioinformatics, Medical Faculty, Heinrich Heine University, Düsseldorf, Germany
[3] Department of Biological Sciences, Middle East Technical University University, Ankara, Turkey
[4] Department of Health Informatics, Graduate School of Informatics, Middle East Technical University University, Ankara, Turkey
[5] Department of Computer Engineering, Bilkent University, Ankara, Turkey

1. Command Lines

1.1. Simulations

1.1.1. Varsim [1]

```
./varsim.py --vc_in_vcf tests/quickstart_test/21_5_10Mb.vcf.gz --sv_insert_seq
insert_seq.txt --sv_dgv GRCh37_hg19_supportingvariants_2013-07-23.txt
--reference $REFERENCE_GENOME --id varsimu --sv_num_del 2000
--sv_num_dup 2000 --sv_percent_novel 0.01 --sv_min_length_lim 1000
--sv_max_length_lim 10000 --total_coverage 10 --java_max_mem 50g
--simulator_executable art_bin_GreatSmokyMountains/art_illumina
```

1.1.2. Gargammel [2]

```
perl gargammel.pl -c $COVERAGE --comp 0,0,1 -damage 0.024,0.36,0.0097,0.55 -f
src/sizefreq.size.gz -s /usr/local/sw/gargammel/src/sizedist.size.gz -ss HS25 -o
$OUTPUT
```

1.1.3 Adapter Removal and Merge [3]

```
AdapterRemoval --file1 $LEFT_PAIR --file2 $RIGHT_PAIR --qualitybase 33 --gzip
--qualitymax 60 --trimns --collapse --minalignmentlength 11 --basename $FILENAME
--settings $FILENAME.settings

cat $FILENAME.collapsed.gz $FILENAME.collapsed.truncated.gz
$FILENAME.pair1.truncated.gz
$FILENAME.pair2.truncated.gz>$FILENAME.all.fastq.gz
```

1.2. Alignment

Alignment to the reference genome [4]**:**

```
bwa aln -l 16500 -n 0.01 -o 2 -t ${cores} ${REFERENCE_GENOME}
${mergedname} \
| $bwa samse ${REFERENCE_GENOME} - ${mergedname} \
| samtools view -F 4 -h -Su - \
| samtools sort - -o ${alndir}/mapped/${filebase}.${refbase}.bam
```

Filter out PCR duplicates (FilterUniqueSAMCons_rand.py) [5]**:**

```
samtools view -F 4 -h ${alndir}/mapped/${filebase}.${refbase}.bam \
| python ${scriptdir}/FilterUniqueSAMCons_rand.py | samtools view -h -Su - \
> ${alndir}/mapped/${filebase}.${refbase}.cons.bam
```

Filter out high mismatch and short reads (percidentity_threshold.py):

```
samtools calmd ${alndir}/mapped/${filebase}.${refbase}.cons.bam
${REFERENCE_GENOME} \
| python ${scriptdir}/percidentity_threshold.py 0.9 35 ${TMPDIR}/short.txt \
| samtools view -bS - > ${alndir}/${filebase}.${refbase}.cons.90perc.bam
```

1.3. CNV Discovery and Genotyping

CONGA:

```
conga -i $BAM_FILE --sonic $SONIC_FILE --ref $REFERENCE_GENOME
--dels $DELS_INPUT --dups $DUPS_INPUT --mappability
$MAPPABILITY_FILE -o $OUTPUT
```

CNVNator [6]**:**

```
cnvnator -root file.root -tree $BAM_FILE -chrom 1 2 3 4 5 6 7 8 9 10 11 12 13 14 15 16 17 18 19 20 21 22 X Y MT

cnvnator -root file.root -his 1000 -d /ref/

cnvnator -root file.root -stat 1000

cnvnator -root file.root -partition 1000

cnvnator -root file.root -call 1000 >$OUTPUT
```

* Note that we used window of 100 for BAM files with small variations and 1000 in all the other BAMs.

FREEC [7]**:**

```
freec -conf data/config_WGS_human.txt
```

* Default configurations were used in the config file of FREEC

GenomeSTRiP [8]**:**

```
java -cp $SV_CLASSPATH ${javaMaxMemory} \
org.broadinstitute.gatk.queue.QCommandLine \
-S ${SV_DIR}/qscript/SVPreprocess.q \
-S ${SV_DIR}/qscript/SVQScript.q \
-configFile ${SV_DIR}/conf/genstrip_parameters.txt \
-tempDir tmp \
-gatk ${SV_DIR}/lib/gatk/GenomeAnalysisTK.jar \
-cp ${SV_CLASSPATH} \
-jobLogDir metadata/logs \
-R $REFERENCE_GENOME \
-md metadata \
-ploidyMapFile ${ploidy} \
-I ${bamFileList} \
-genomeMaskFile  $REFERENCE_GENOME_MASK36 \
-copyNumberMaskFile  $REFERENCE_GENOME_CN2_MASK \
-bamFilesAreDisjoint true \
-computeGCProfiles true \
-deleteIntermediateDirs true \
```

```
-jobRunner ParallelShell \
-run \

java -cp ${SV_CLASSPATH} -Xmx4g \
org.broadinstitute.gatk.queue.QCommandLine \
-S ${SV_DIR}/qscript/SVGenotyper2.q \
-S ${SV_DIR}/qscript/SVQScript.q \
-gatk ${SV_DIR}/lib/gatk/GenomeAnalysisTK.jar \
-cp ${SV_CLASSPATH} \
-configFile ${SV_DIR}/conf/genstrip_parameters.txt \
-jobLogDir ${runDir}/logs \
-tempDir ${SV_TMPDIR} \
-R ${referenceFile} \
-md ${mdPath} \
-genomeMaskFile $REFERENCE_GENOME_MASK36 \
-genderMapFile ${SV_DIR}/input/sample_gender.report.txt \
-I ${bamFileList} \
-parallelRecords 1000 \
-runDirectory ${runDir} \
-vcf ${vcfFile} \
-skipAnnotator CNQuality \
-O /${runDir}/output_file.genotypes.vcf \
-jobRunner ParallelShell \
-gatkJobRunner ParallelShell \
-run
```

* We used default mask files for human genome grch37 with k-mer size 36.

mrCaNaVaR

Alignment using mrsFAST [9]:

```
mrsfast --search $MASKED_REFERENCE_GENOME --seq $FASTQ_FILE
--seqcomp --threads 24 -o sim.sam --disable-nohits --crop 36
```

Variant Calling using mrCaNaVaR [10, 11]**:**

```
mrcanavar --read -conf human_g1k_v37.cnvr -samlist $SAM_FILE -depth
$DEPTH_FILE_NAME

mrcanavar --call human_g1k_v37.cnvr -depth $DEPTH_FILE_NAME -o
$OUTPUT_FILE
```

1.4. BEDTools [12]

TRUE_PREDICTION = ~/bedtools2/bin/intersectBed -a $TRUE_CALLS -b del.bed -f 0.5 -wa -r|grep -v X|grep -v Y|grep -v GL|grep -v MT|sort -u|wc

FALSE_PREDICTION = ~/bedtools2/bin/intersectBed -a del.bed -b $TRUE_CALLS -f 0.5 -r -wa -v|grep -v GL|grep -v X|grep -v Y|grep -v MT|sort -u|wc

FDR = FALSE_PREDICTION / (TRUE_PREDICTION + FALSE_PREDICTION)

RECALL = TRUE_PREDICTION / (TRUE_PREDICTION + FALSE_PREDICTION)

PRECISION = TRUE_PREDICTION / TRUE_CALLS

F-SCORE = (2 * PRECISION * RECALL) / (PRECISION + RECALL)

1.5 Depth of coverage calculation [13]

```
samtools view -b -q 30 -F 4 XXX.bam | genomeCoverageBed -ibam - -g human_g1k_v37.fasta >coverage.cov

grep genome coverage.cov | awk '{NUM+=$2*$3; DEN+=$3} END {print NUM/DEN}'
```

1.6 Down-sampling

```
java -jar ~/picard.jar DownsampleSam I=XXX.bam O=XXX_0004.bam P=0.004
```

* In our experiments, we used “P” values of 0.7, 0.3, 0.1, 0.05, 0.03, 0.01, 0.04, 0.03 for Yamnaya, 0.7, 0.3, 0.1, 0.05, 0.03, 0.01, 0.04 for Saqqaq and 0.7, 0.3, 0.1, 0.05, 0.03 for Mota genomes.

2. Supplemental Notes

Supplemental Note 1:

The vast majority of structural variant studies (>92%) available in the European Variation Archive (EVA) are focused on humans (as of March 2022), while the NCBI dbVar has stopped storing non-human SVs. Nevertheless, the EVA database compiles all types of genetic variation data including CNVs from species ranging from the chimpanzee, gorilla, orangutan, rhesus macaque, dog, cow, horse, pig, mouse, zebrafish, and sorghum [14]. Furthermore, CNVs for other organisms are also available in specific databases such as those for *Arabidopsis* [15] and the *Anopheles* mosquito [16].

Supplemental Note 2:

Here we discuss the reasons for CONGA's under-performance in genotyping duplications in down-sampling experiments. CONGA's accuracy was particularly low in trials using the Yamnaya genome. This was despite high performance in duplication estimation with simulated genomes. Studying the results in further detail, we noticed that only 47 (2.8%) of the 1,661 originally called duplication events in the full Yamnaya genome were genotyped using read-depth information (i.e. the C-score), while the rest were genotyped using paired-end read information. In contrast, 35% (1498/4027) and 27% (172/638) of duplication events were genotyped using read-depth information on the full Saqqaq and Mota genomes, respectively.

This difference, in turn, can explain CONGA's under-performance at low coverage in the Yamnaya genome: As coverage decreases, the number of paired-end reads supporting a duplication falls rapidly, compromising recall. Meanwhile, the lack of usable read-depth information in the full Yamnaya genome could be related to alignment quality filters applied to the BAM file before data publication. Such filtering likely erased the read-depth signature, leaving only paired-end information available, which is not helpful at low coverages. This scenario is supported by the fact CONGA duplication calls are clearly more successful in the Saqqaq and Mota genomes at low coverage. These two were retrieved as original FASTQ files instead of processed BAM files. The results underscore the need for publishing raw FASTQ files rather than BAM files to allow healthy reuse of the data.

Supplemental Note 3:

Here we describe how we selected the divergent, or outlier genome set. We genotyped n=71 real ancient genomes from 10 different laboratories, using all 10,002 human-derived deletions identified in the 1000 Genomes Project African dataset. The presence of technical effects is suggested by a marginally significant laboratory-of-origin effect on deletion frequencies per genome in this set (Kruskal-Wallis test p=0.08). We therefore created heatmaps, multidimensional scaling plots summarizing Euclidean distance matrices, hierarchical clustering trees summarizing Manhattan distance matrices, and principal

component analysis plots (PCA) summarizing deletion frequencies (the latter after removing any NAs). We intentionally used a variety of methods in order to capture different potential sources of variability. The results suggested outlier behavior across n=11 genomes, some that we observed to have relatively low coverage (e.g. ne4, ko2, MA1, DA379), were produced with the capture protocol (Bon002), or originated from the same laboratory despite having variable population origins (e.g. R1, R2, R3, R4, R7, R9; which include Mesolithic and Neolithic genomes) (also see S2 Table). Removing these 11 genomes and repeating the analysis with the remaining 60, we again found a significant laboratory-of-origin effect on deletion frequencies per genome (Kruskal-Wallis test p=0.02). The PCA, MDS and hierarchical clustering analyses on the 60 genomes again suggested the presence of a number of divergent cases, mainly from the same laboratories (Iceman, RISE493, RISE495, RISE496, RISE505, RISE511, SI.38, SI.41, SI.45, SI.53). The genome Chan (a European Mesolithic individual) also showed divergent behavior in the PCA, but this genome has been previously identified as being highly homozygous [17], and it was in fact close to other European Mesolithic individual genomes in its deletion profile, suggesting that the outlier behavior may be related to its excess homozygosity (indeed this genome had the highest number of homozygous deletions among the 50). We therefore did not remove Chan from the set. We recommend similar quality controls on genotyped deletion datasets created from heterogeneous ancient genomes.

Supplemental Note 4:

Here we describe how we make use of split-read and paired-end signatures for duplication genotyping

Beyond read-depth, information of paired-end reads or read fragments that do not linearly map to the genome can be used to identify CNVs. Ancient genomes are sometimes single-end and sometimes paired-end sequenced, but in the latter case, short overlapping reads are typically merged into a single read before alignment. Ancient genome data is thus practically single-read. However, the split-read method can be applied on single-read ancient genome data, which emulates paired-end information for genotyping duplications. This approach is visualized in S14 Fig. We therefore designed CONGA to include both paired-end and single-end reads as input and to evaluate paired-end signature information.

First, assume a read of length L mapped to position *pos_x* in the reference genome, where *pos_x* is assumed to be one of the breakpoints of a putative CNV. There always exists a subsequence $\geq L/2$ that will have at least one mapping in the reference genome with some error threshold. Thus, we can split a read into two subsequences, assigning the actual mapping to one of the pairs and remapping the other subsequence ("split segment") as a second pair. There are two possible split strategies: an even decomposition, where both subsequences are of equal lengths, or an uneven decomposition, where the subsequences are of unequal lengths. Given the infeasibility of testing each split position and the fact that ancient reads are typically already short, we follow [18] and split the read from the middle to obtain two reads with equal lengths $L/2$. If a read overlaps a duplication breakpoint, and assuming that the expected position of the breakpoint will be uniformly distributed within the read, the split segment will map to the reference genome with insert size -the distance between the split-read pairs- greater than zero.

With this simple observation, the need to observe all possible breakpoints can be eliminated. Thus, given a single-end read Rse_i, we define Rpe_i = (l(Rpe_i [pos_x : pos_x + *RL*/2]) and r(Rpe_i [pos_y : pos_y + *RL*/2])), where pos_x is the initial mapping position of the single-end read, pos_y is the remapping position of the split read, *RL* is the length of the single-end read observed before the split, l(Rpe_i [pos_x : pos_x + *RL*/2]) is the left pair within pos_x and pos_x + *RL*/2 and r(Rpe_i [pos_y : pos_y + *RL*/2]) is the right pair within pos_y and pos_y + *RL*/2 of the paired-end reads.

According to our remapping strategy, we use a seed-and-extend approach similar to that implemented in mrFAST [19], where a read is allowed to be mapped to multiple positions. Our main concern here is that the split segment, due to its short length, can be mapped to unrealistically high numbers of positions across the genome. To overcome this problem we use the approach developed in TARDIS [20], allowing the split segment to be mapped only up to 10 positions within close proximity (15 kbps by default) of the original mapping position and applying a Hamming distance threshold for mismatches (5% of the read length by default).

Based on the distance between the reads (insert-size) and orientation, we then evaluate the type of putative CNV. As S15 Fig shows, if the split segment maps behind the initially mapped segment of the same pair to generate a reverse-forward mapping orientation, this would be an indication of a duplication.

In order to utilize this paired-read information, for each CNV locus used as input to our algorithm, we count the number of read-pair (i.e. split segments) that map around +/- 5 kbps of the breakpoints. Each such read-pair is treated as one observation. We use these counts in combination with the C-score (read-depth information) to genotype duplications (see Methods Section). We do not use this read-pair information for genotyping deletions due to its low effectiveness in our initial trials (Table E in S1 Table).

References

1. Mu JC, Mohiyuddin M, Li J, Bani Asadi N, Gerstein MB, Abyzov A, et al. VarSim: a high-fidelity simulation and validation framework for high-throughput genome sequencing with cancer applications. Bioinformatics. 2015 May 1;31(9):1469–71.
2. Renaud G, Hanghøj K, Willerslev E, Orlando L. gargammel: a sequence simulator for ancient DNA. Bioinformatics. 2016 Oct 29;btw670.
3. Schubert M, Lindgreen S, Orlando L. AdapterRemoval v2: rapid adapter trimming, identification, and read merging. BMC Res Notes. 2016 Dec;9(1):88.
4. Li H, Durbin R. Fast and accurate short read alignment with Burrows-Wheeler transform. Bioinformatics. 2009 Jul 15;25(14):1754–60.
5. Kircher M. Analysis of High-Throughput Ancient DNA Sequencing Data. In: Shapiro B, Hofreiter M, editors. Ancient DNA [Internet]. Totowa, NJ: Humana Press; 2012 [cited 2021 Nov 9]. p. 197–228. (Methods in Molecular Biology; vol. 840). Available from: http://link.springer.com/10.1007/978-1-61779-516-9_23
6. Abyzov A, Urban AE, Snyder M, Gerstein M. CNVnator: An approach to discover, genotype, and characterize typical and atypical CNVs from family and population genome sequencing. Genome Res. 2011 Jun 1;21(6):974–84.
7. Boeva V, Popova T, Bleakley K, Chiche P, Cappo J, Schleiermacher G, et al. Control-FREEC: a tool for assessing copy number and allelic content using next-generation sequencing data. Bioinformatics. 2012 Feb 1;28(3):423–5.
8. Handsaker RE, Korn JM, Nemesh J, McCarroll SA. Discovery and genotyping of genome structural polymorphism by sequencing on a population scale. Nat Genet. 2011 Mar;43(3):269–76.
9. Hach F, Hormozdiari F, Alkan C, Hormozdiari F, Birol I, Eichler EE, et al. mrsFast: a cache-oblivious algorithm for short-read mapping. 2011;4.
10. Alkan C. Automatic characterization of copy number polymorphism using high throughput sequencing. :9.
11. Kahveci F, Alkan C. Whole-Genome Shotgun Sequence CNV Detection Using Read Depth. In: Bickhart DM, editor. Copy Number Variants [Internet]. New York, NY: Springer New York; 2018 [cited 2022 Mar 21]. p. 61–72. (Methods in Molecular Biology vol.1833). Available from: http://link.springer.com/10.1007/978-1-4939-8666-8_4
12. Quinlan AR, Hall IM. BEDTools: a flexible suite of utilities for comparing genomic features. Bioinformatics. 2010 Mar 15;26(6):841–2.
13. Li H, Handsaker B, Wysoker A, Fennell T, Ruan J, Homer N, et al. The Sequence Alignment/Map format and SAMtools. Bioinformatics. 2009 Aug 15;25(16):2078–9.
14. Cezard T, Cunningham F, Hunt SE, Koylass B, Kumar N, Saunders G, et al. The European Variation Archive: a FAIR resource of genomic variation for all species. Nucleic Acids Res. 2022 Jan 7;50(D1):D1216–20.
15. Zmienko A, Marszalek-Zenczak M, Wojciechowski P, Samelak-Czajka A, Luczak M, Kozlowski P, et al. AthCNV: A Map of DNA Copy Number Variations in the Arabidopsis Genome. Plant Cell. 2020 Jun;32(6):1797–819.
16. Lucas ER, Miles A, Harding NJ, Clarkson CS, Lawniczak MKN, Kwiatkowski DP, et al. Whole-genome sequencing reveals high complexity of copy number variation at insecticide resistance loci in malaria mosquitoes. Genome Res. 2019 Aug;29(8):1250–61.
17. Ceballos FC, Gürün K, Altınışık NE, Gemici HC, Karamurat C, Koptekin D, et al. Human

inbreeding has decreased in time through the Holocene. Curr Biol. 2021 Sep;31(17):3925-3934.e8.
18. Karakoc E, Alkan C, O'Roak BJ, Dennis MY, Vives L, Mark K, et al. Detection of structural variants and indels within exome data. Nat Methods. 2012;9(2):176–8.
19. Alkan C, Kidd JM, Marques-Bonet T, Aksay G, Antonacci F, Hormozdiari F, et al. Personalized copy number and segmental duplication maps using next-generation sequencing. Nat Genet. 2009 Oct;41(10):1061–7.
20. Soylev A, Kockan C, Hormozdiari F, Alkan C. Toolkit for automated and rapid discovery of structural variants. Methods. Oct 1;129:3–7.
