# Supplementary figures and images for: CONGA: Copy number variation genotyping in ancient genomes and low-coverage sequencing data
Source: PLoS Comput Biol. 2022 Dec 14;18(12):e1010788. doi: 10.1371/journal.pcbi.1010788 (PMC9873172; doi:10.1371/journal.pcbi.1010788)

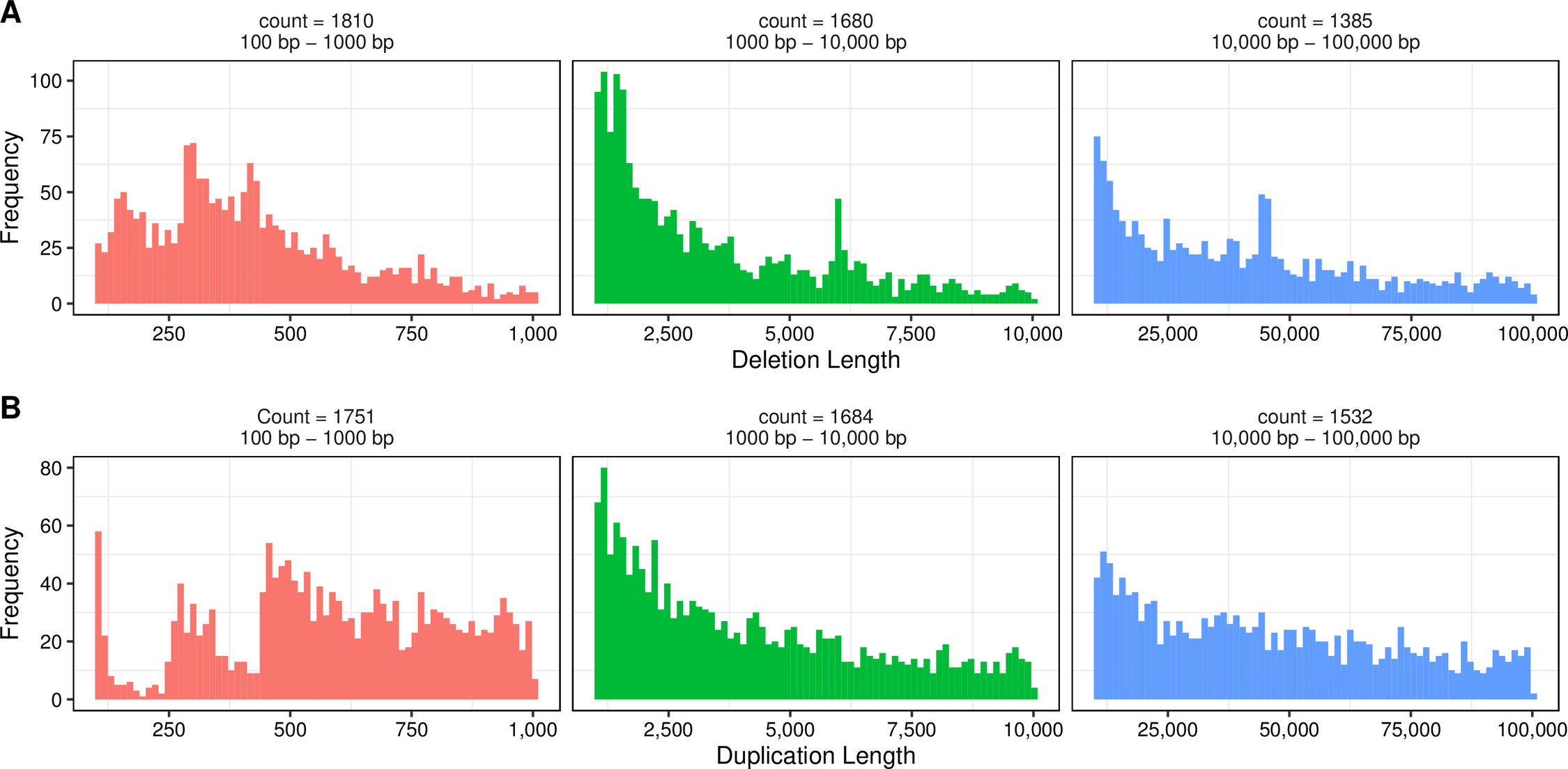

Supplement: S1 Fig — The total number of CNVs inserted into a genome (“counts”) is shown at the top of each graph. We used Varsim to insert these CNVs into each genome, yielding three genomes in total (for short, medium and large CNVs). (TIF) [file pcbi.1010788.s002.tif]

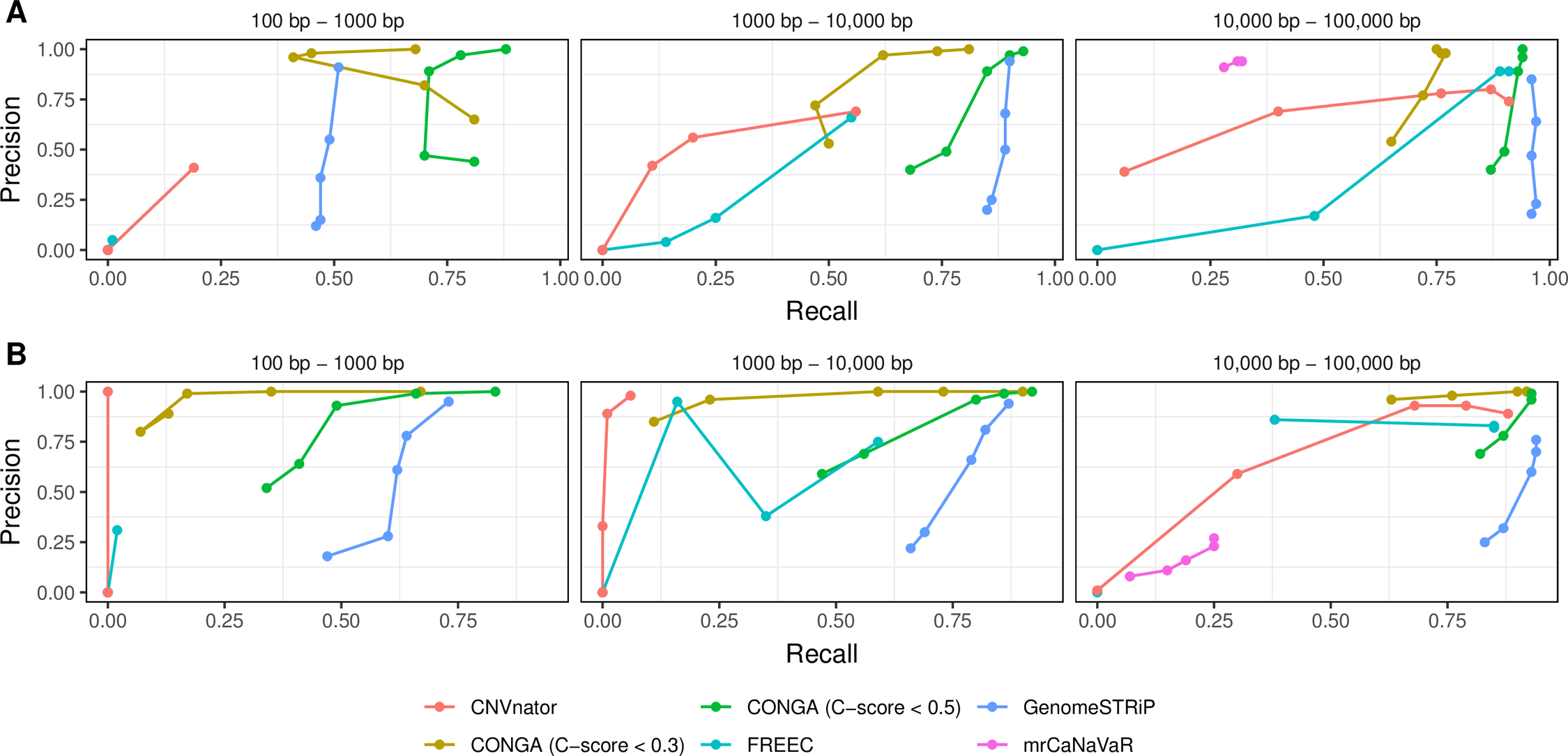

Supplement: S2 Fig — Precision-Recall curves for deletion (A) and duplication (B) predictions of CONGA, GenomeSTRiP, FREEC, and CNVnator using coverages of 0.05×, 0.1×, 0.5×, 1× and 5×. mrCaNaVaR was used only in the analysis of large variants. (TIF) [file pcbi.1010788.s003.tif]

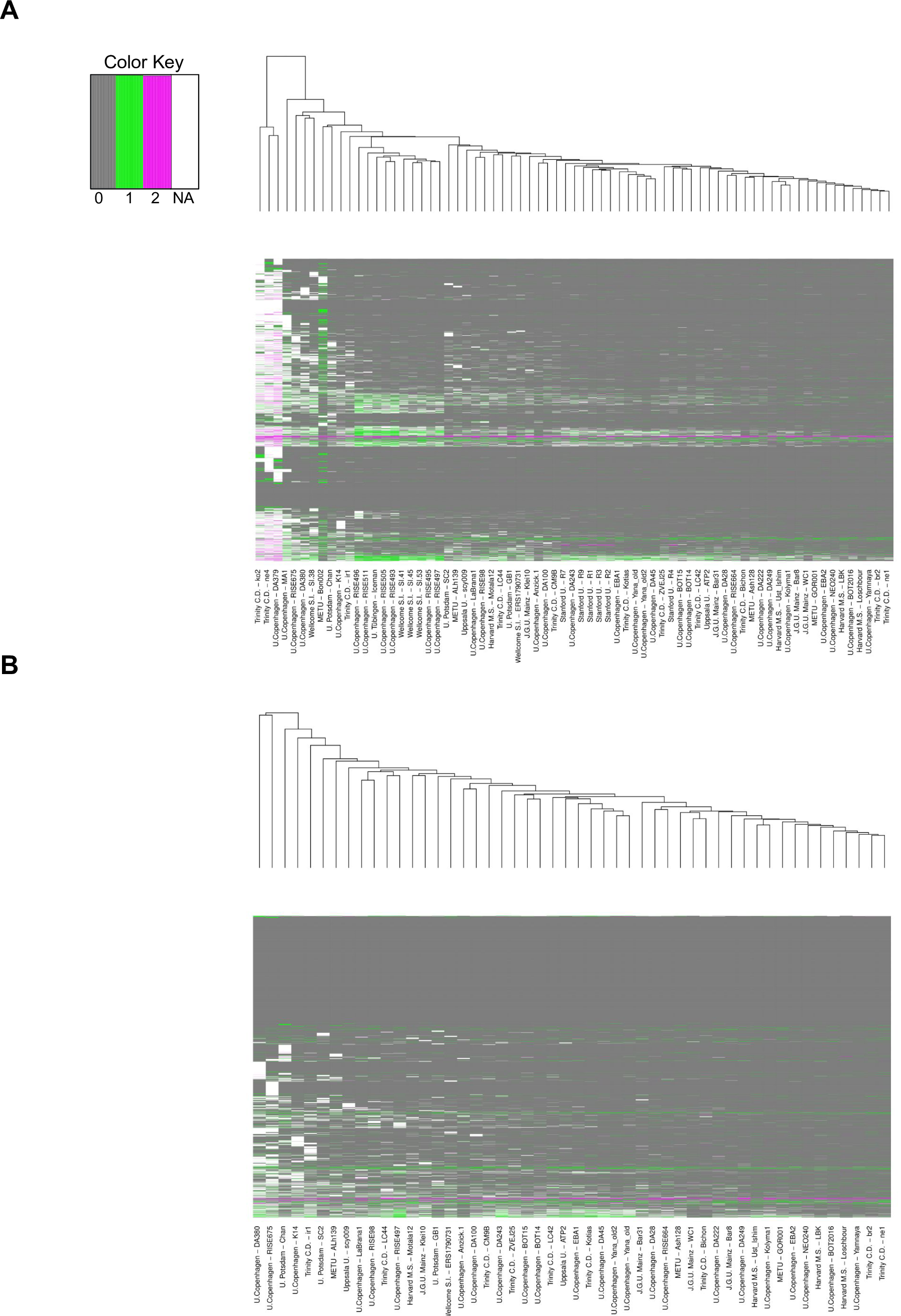

Supplement: S3 Fig — The color key includes 0 (gray) for reference allele, 1 (green) for heterozygous, 2 (magenta) for homozygous state and NA (white) for missing value. (A) Heatmap of deletions per genome on the raw dataset (n = 71 genomes). (B) Heatmap of deletions per genome on the refined dataset (with n = 50 genomes after removing divergent genomes). (TIF) [file pcbi.1010788.s004.tif]

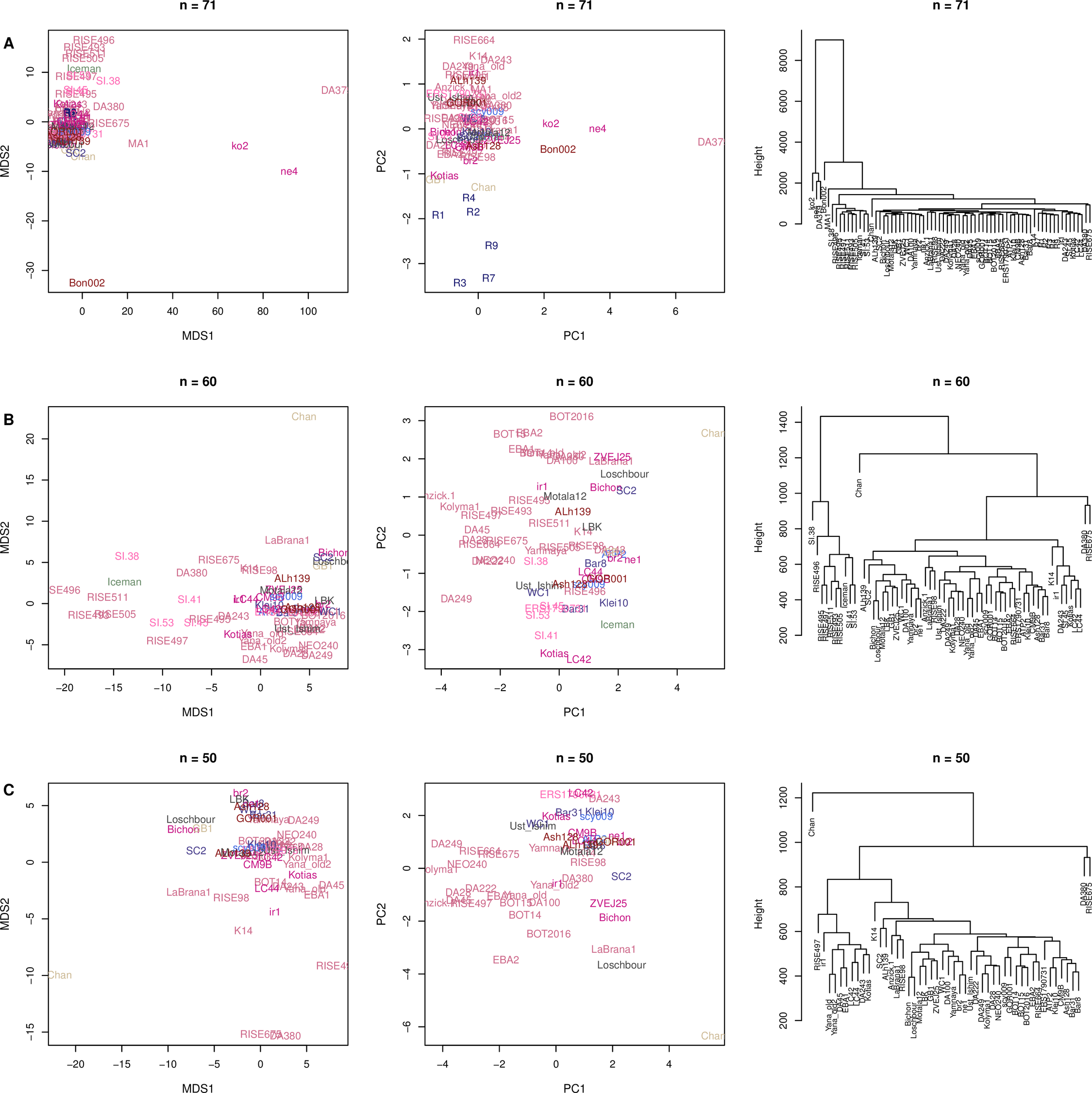

Supplement: S4 Fig — Left panels: Multidimensional scaling plots (MDS) calculated with k = 2 using the R “cmdscale” function on a Euclidean distance matrix of deletion frequencies. Middle panels: Principal component analysis plots (PCA) summarizing deletion frequencies after removing any NAs. Right panels: Hierarchical clustering trees summarizing Manhattan distance matrices, calculated using the R “dist” and “hclust” functions. The color codes indicate the laboratory-of-origin of each genome, shown in the legend of the top right panel. (A) Results based on the full dataset with 10,002 human-derived deletions (n = 8,780 genotyped in any state in at least one genome) and n = 71 genomes. In the PCA we use nD = 580 deletions after removing loci with at least one missing value. (B) Results based on n = 60 genomes after removing 11 outlier genomes (and nD = 3,460 deletions in the PCA). (C) Results based on n = 50 genomes after removing 21 outlier genomes (and nD = 3,472 deletions in the PCA). We note that the MDS here differs from that shown in Fig 5, in that the latter is calculated using outgroup-f3 statistics. (TIF) [file pcbi.1010788.s005.tif]

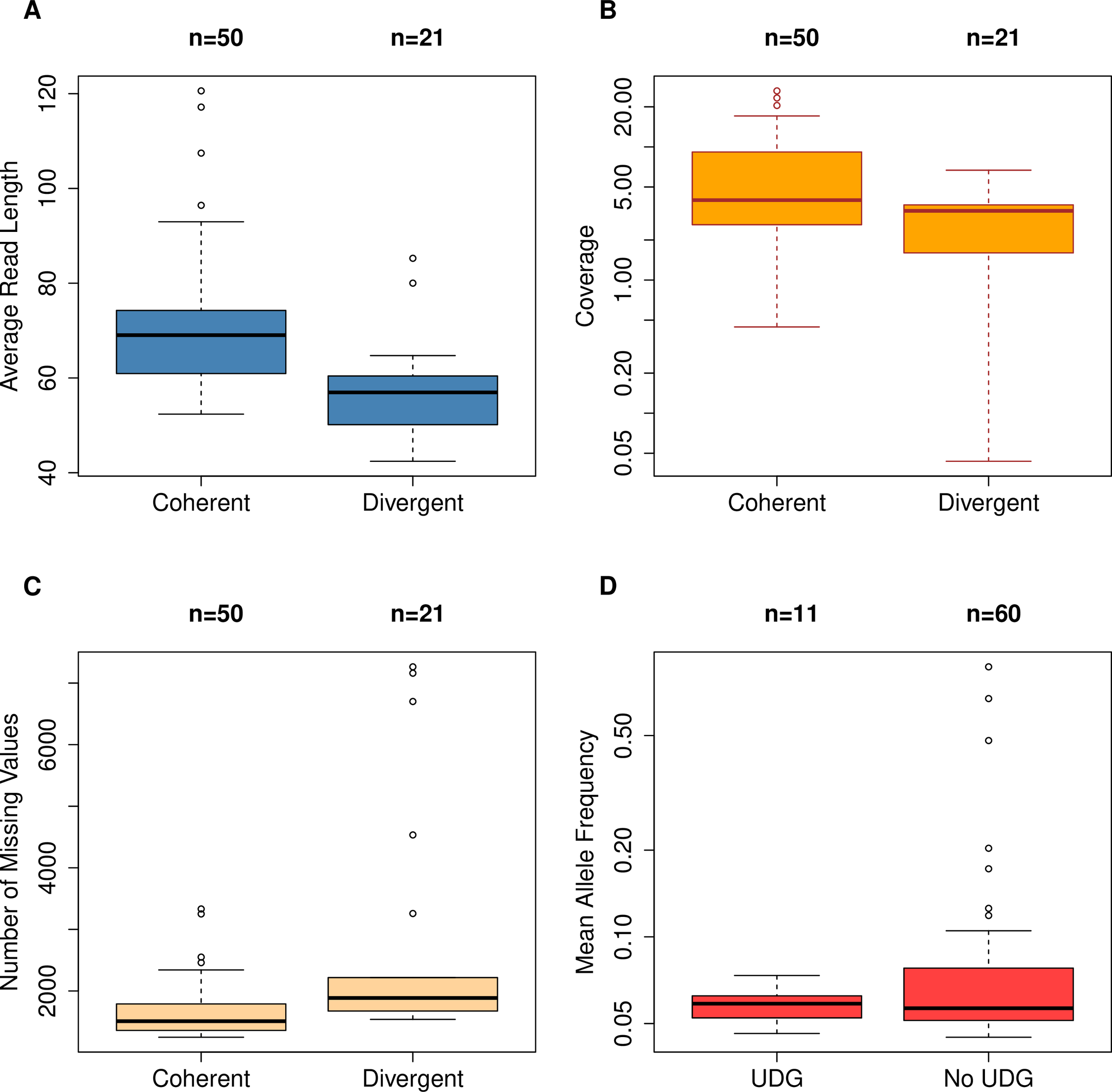

Supplement: S5 Fig — This is defined based on their deletion profiles (S3 and S4 Figs) (A) Boxplots of the average read length per genome (Wilcoxon-rank sum test, P<0.001). (B) Boxplots of mean depths of coverage (Wilcoxon rank sum test, P = 0.014). (C) Boxplots of the number of missing values per genome (Wilcoxon-rank sum test, P<0.001). (D) Boxplots of the mean deletion allele frequencies per UDG-treated and not UDG-treated genomes. We observe no significant difference between the distributions (Wilcoxon-rank sum test, P = 0.58). (TIF) [file pcbi.1010788.s006.tif]

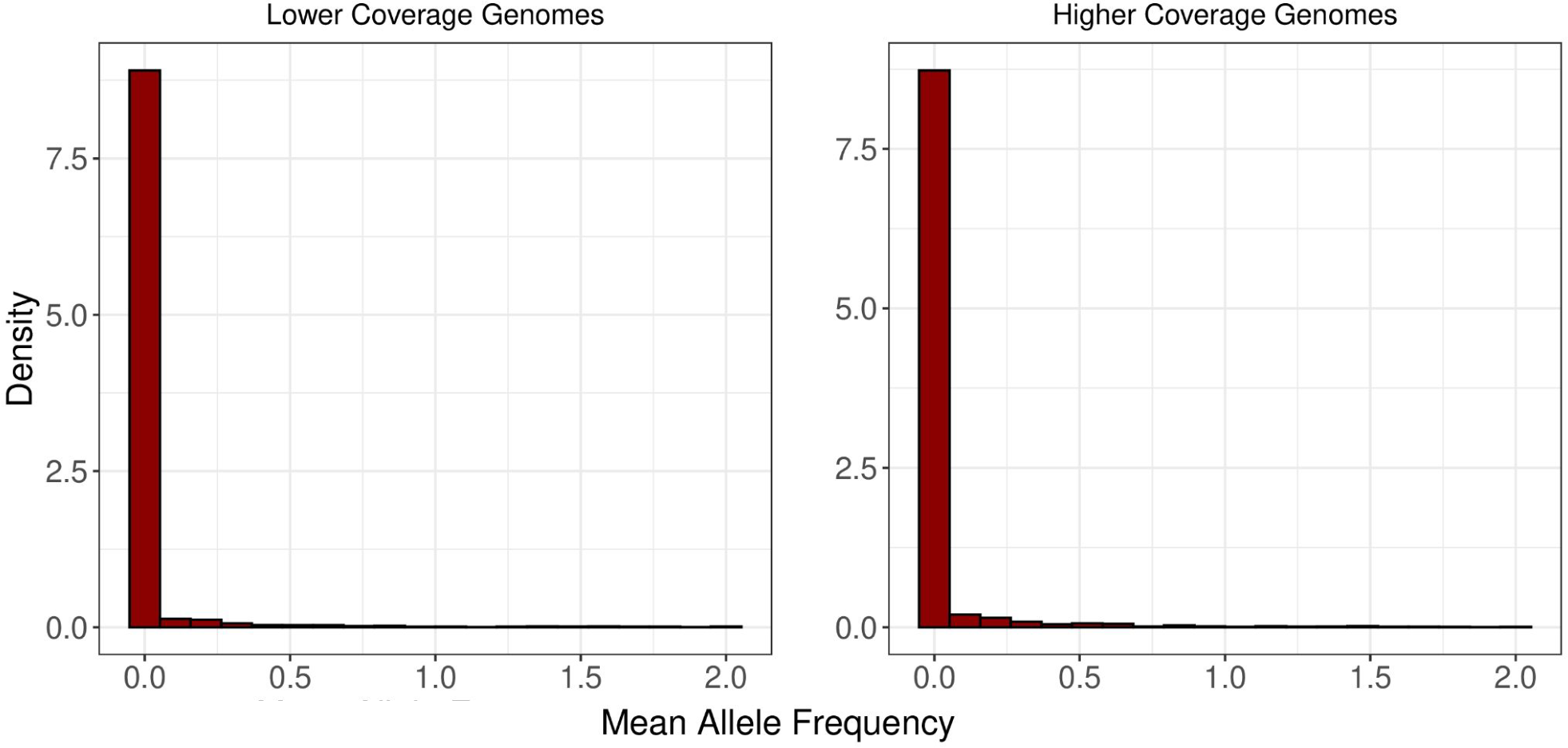

Supplement: S6 Fig — Left panel represents the SFS of n = 25 below-median coverage genomes and right panel shows the SFS of n = 25 above-median coverage genomes. The median coverage value was 3.98×. We found no significant difference between the two SFS distributions (Kolmogorov-Smirnov test ρ = 0.27). (TIF) [file pcbi.1010788.s007.tif]

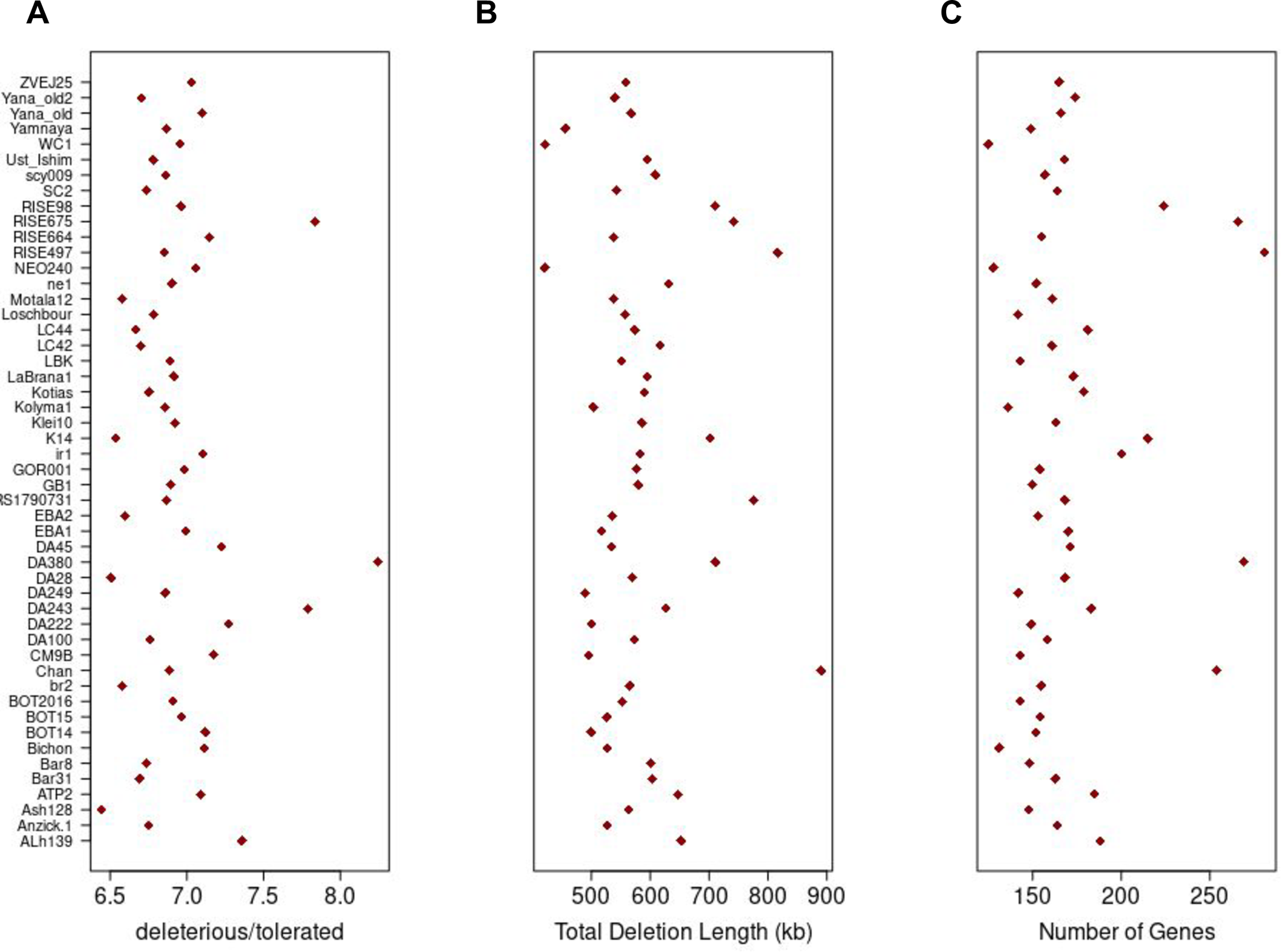

Supplement: S7 Fig — In all three panels, the x-axis represents a deleterious load-related statistic and the y-axis shows the ancient individuals. (A) Deleterious load based on SIFT-estimated SNP effects per individual. The x-axis represents the number of “deleterious” SNPs over the number of “tolerated” SNPs. (B) CONGA-estimated total deletion length in kb per individual, using the Final CNV call-set. (C) The number of genes that overlap with CONGA-estimated deletions. In panels B and C, heterozygous and homozygous calls were counted once. In panel C, the most affected individuals in terms of the number of gene overlaps are RISE497 (Russia, 2nd millennium BCE), DA380 (Turkmenistan, 4th millennium BCE), RISE675 (Russia, 3rd millennium BCE), and Chan (Iberia, 8th millennium BCE). We observed that these individuals were around 50% more affected than the rest. (TIF) [file pcbi.1010788.s008.tif]

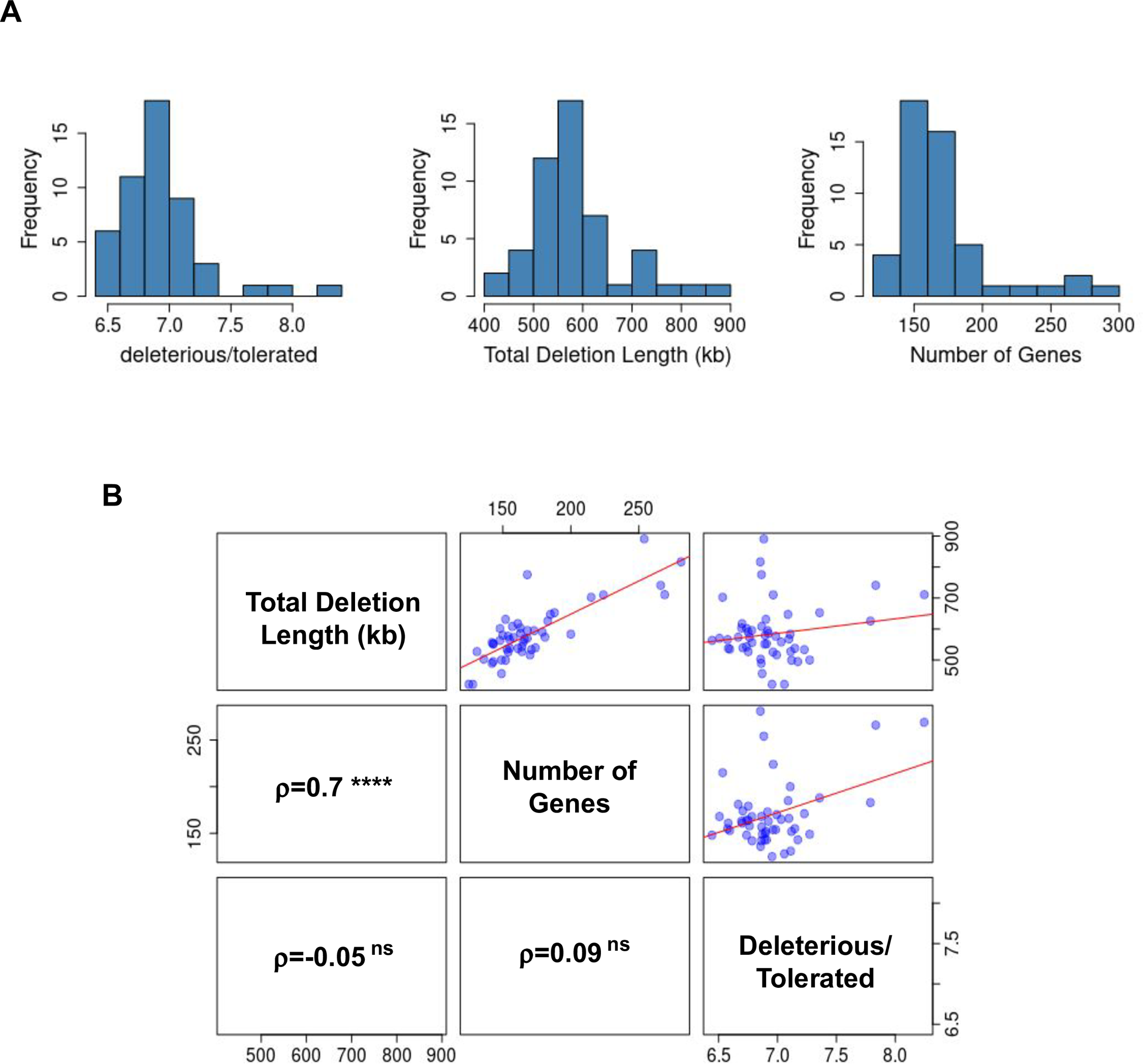

Supplement: S8 Fig — (A) From left to right: histograms of the number of SIFT-predicted “deleterious” SNPs over “tolerated” SNPs per genome, CONGA-predicted total deletion length in kb per genome, and the number of genes that overlap with CONGA-predicted deletions per genome. (B) Correlations between each variable. The RHS triangle shows the scatter plots between two variables, and the LHS triangle shows the Spearman rank correlation estimates. The significance of the ρ’s are also shown. **** ρ<0.0001, ns: non-significant. (TIF) [file pcbi.1010788.s009.tif]

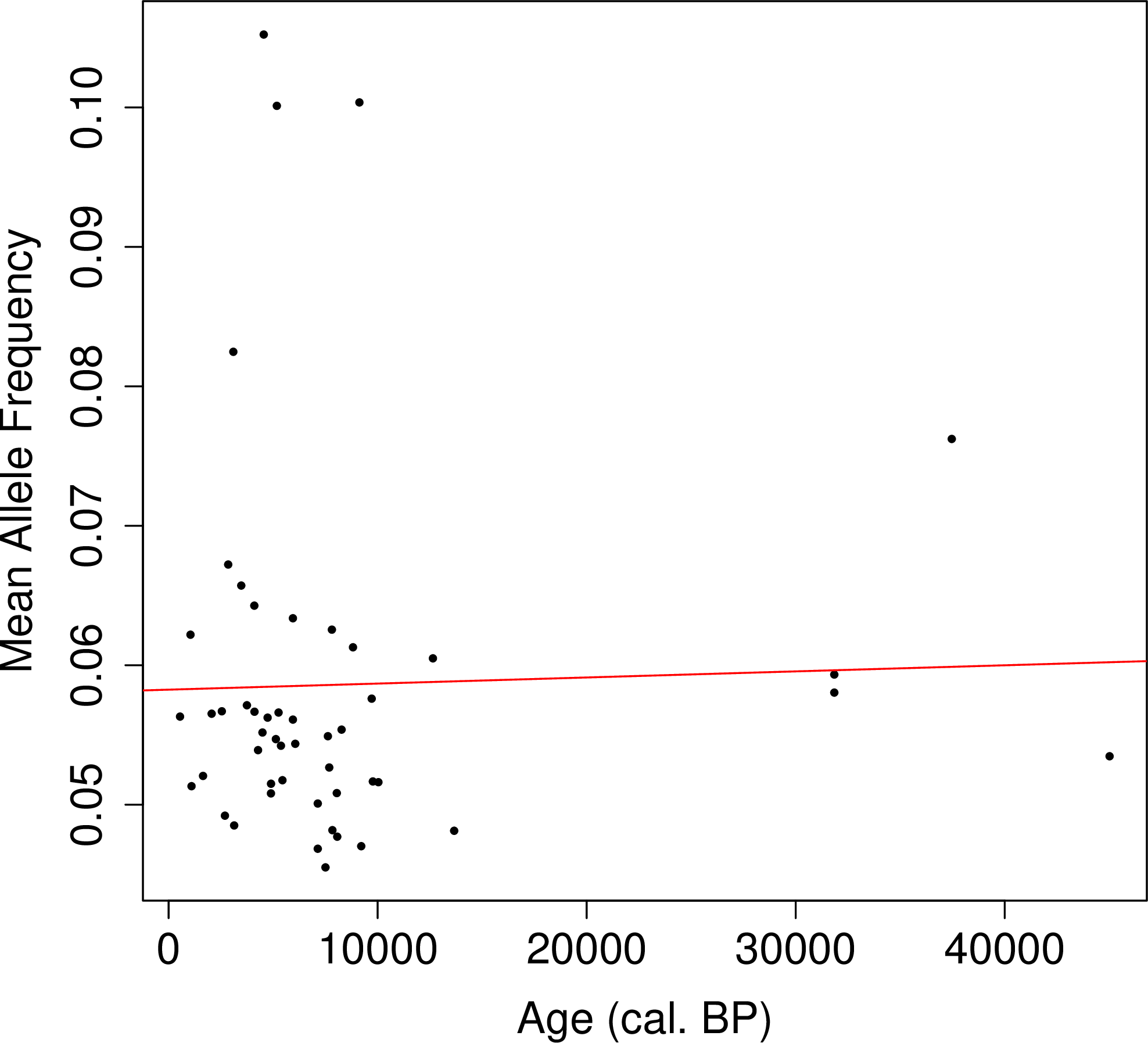

Supplement: S9 Fig — Red line represents the linear model. We found that there is no significant correlation between the age of the individuals and the mean allele frequency (Spearman’s rank correlation ρ = -0.12, P = 0.41). (TIF) [file pcbi.1010788.s010.tif]

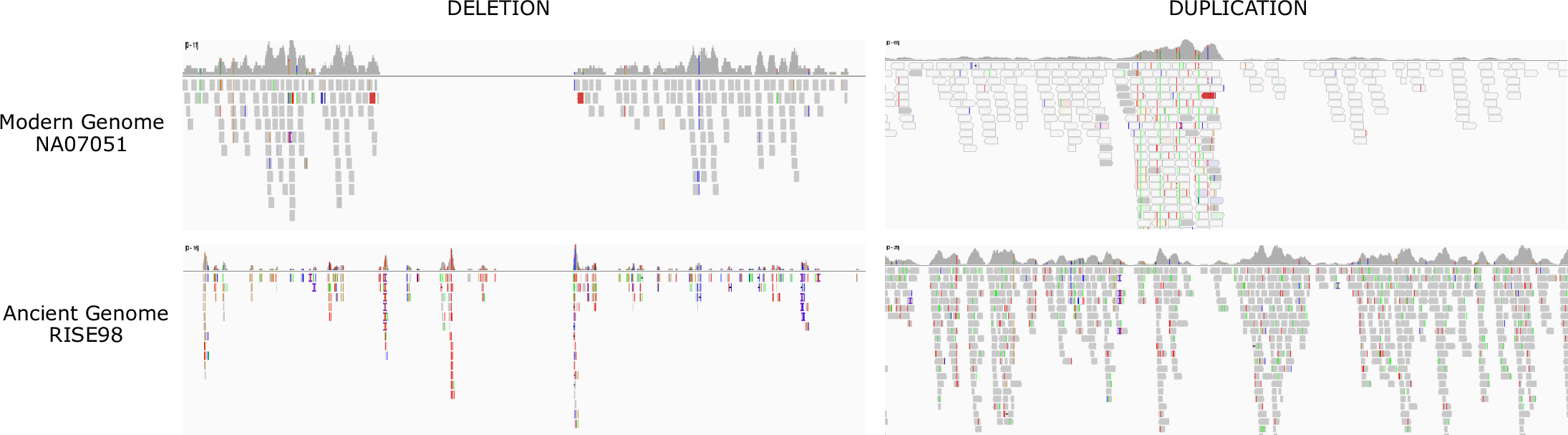

Supplement: S10 Fig — Medium sized CNVs are between 1,000 bps to 10,000 bps and large CNVs are between 10,000 bps and 100,000 bps. Here, we used a relaxed C-score threshold of 10 in order to observe the effect of read-pair support only. The figure shows that read-pair support is effective when the coverage is above 0.5x and also when the duplication sizes are larger. (TIF) [file pcbi.1010788.s011.tif]

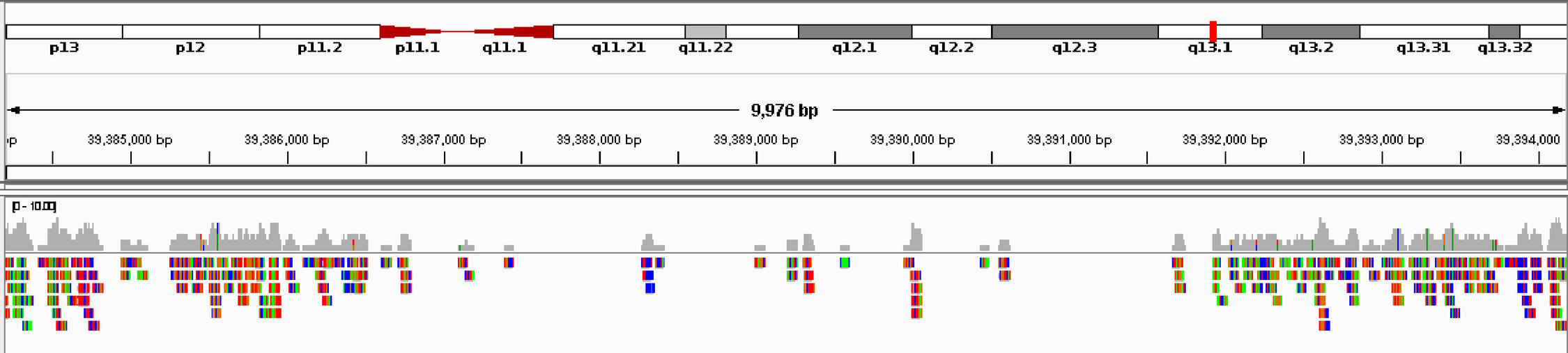

Supplement: S11 Fig — The events displayed in the upper panels were detected in a modern-day human genome (NA07051: an ∼8 kbp deletion within chr7:16,169,440-16,177,556 and a ∼4 kbp duplication within chr7:22,496-26,553) and those in the lower panels in an ancient genome (RISE98: an ∼17 kbp deletion within chr6:32,506,809-32,524,264 and a ∼6 kbp duplication within chr1:1,520,604-1,526,959). The candidate CNV list used for genotyping was the long read CNV dataset described in Methods. Deducing the CNVs is straightforward with the modern-day genome data, however, it is less straightforward to distinguish these variations in ancient read data, especially for duplications. Note that this is one of the sample scenarios and we emphasize that a large number of CNVs identified in ancient genomes suffer from the same issue. (TIF) [file pcbi.1010788.s012.tif]

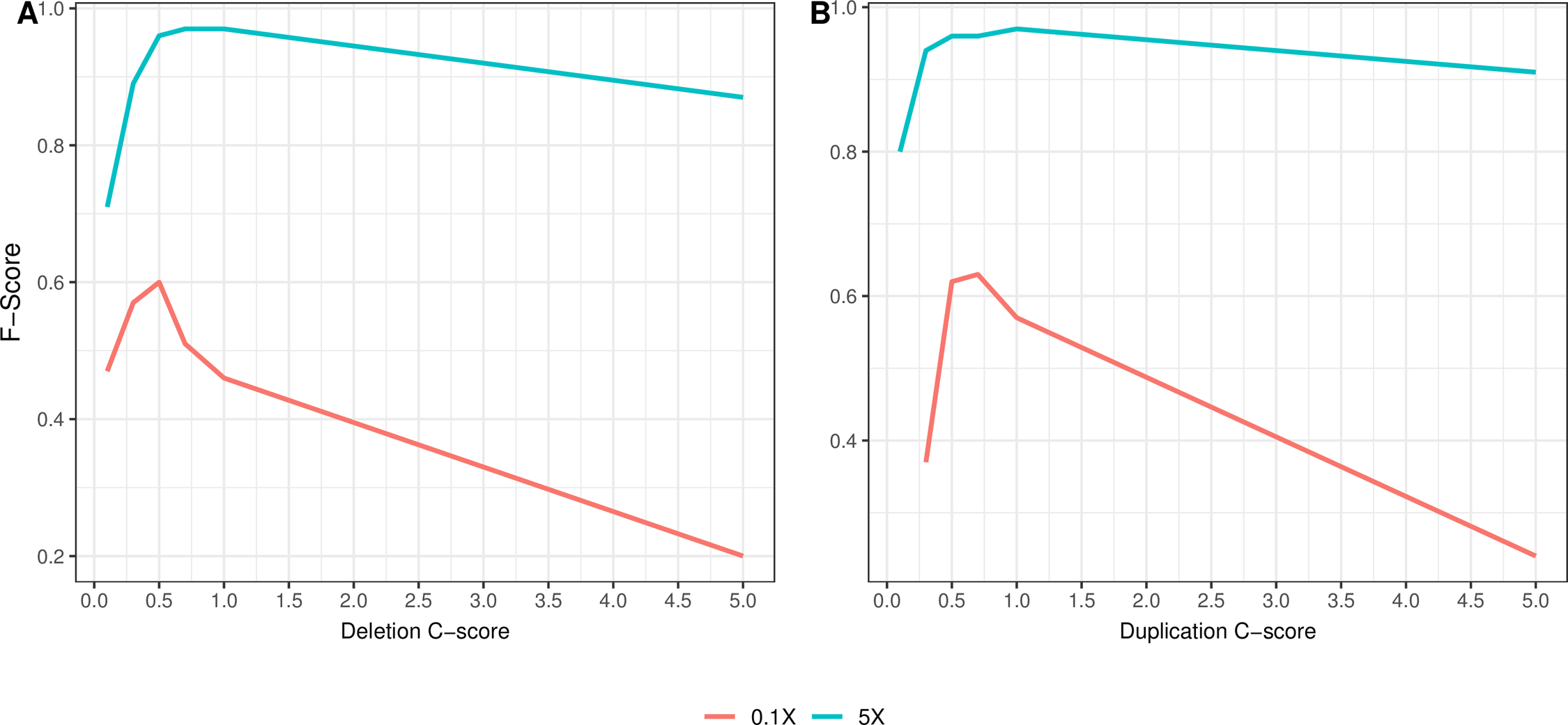

Supplement: S12 Fig — An inserted deletion in a simulated ancient genome at 1× depth of coverage. The event breakpoint is chr22:39,386,521-39,391,930. CONGA missed this deletion due to the poor signal. (TIF) [file pcbi.1010788.s013.tif]

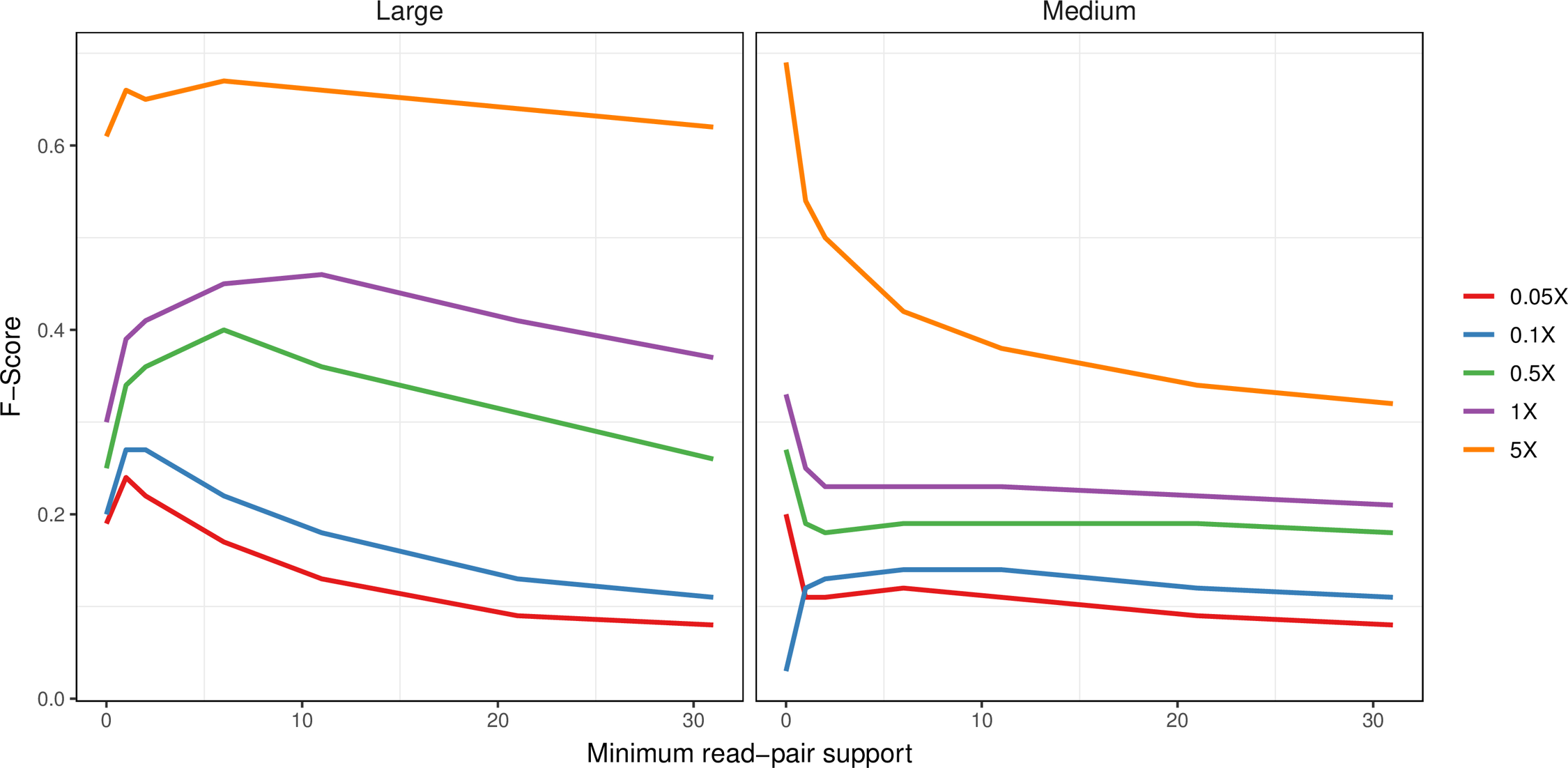

Supplement: S13 Fig — The figure shows the effect of C-score on F-Scores of deletions (A) and duplications (B) for 0.1× and 5× depths of coverages in simulated genomes with medium sized CNVs embedded. Here, we did not use read-pair support or mappability filtering, in order to only test the effect of the C-score threshold. The C-score is calculated using read-depth information. (TIF) [file pcbi.1010788.s014.tif]

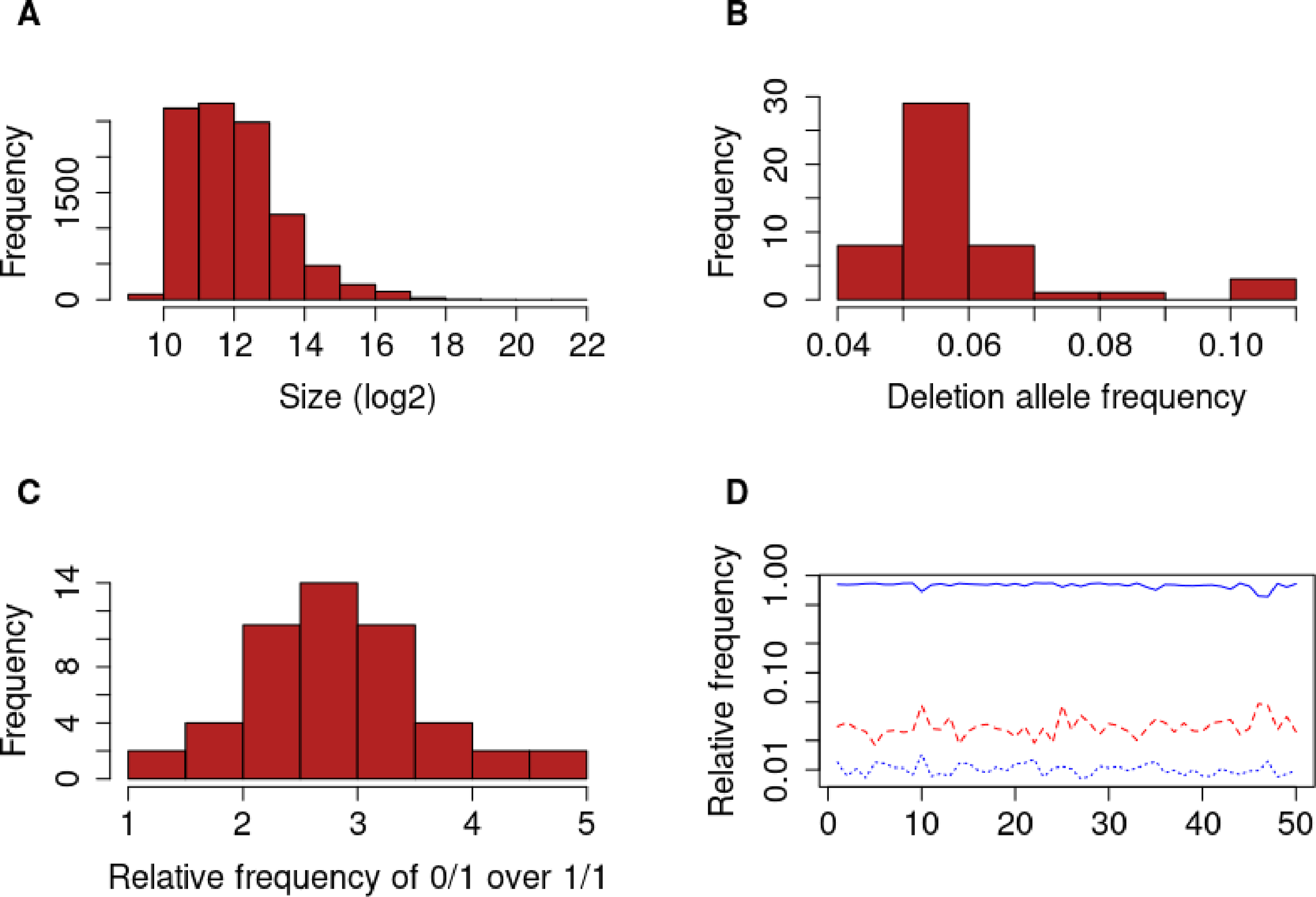

Supplement: S14 Fig — (A) Size distribution of the deletions in logarithmic scale. (B) The distribution of the deletion allele frequency (i.e. the proportion of deletion alleles across the 8,780 loci per genome) among the 50 genomes. (C) The distribution of the relative frequency of observed heterozygous (0/1) deletions over homozygous (1/1) deletions observed in our dataset. (D) The plot of relative deletion frequencies called heterozygous (red lines) and homozygous (blue lines) among 8,780 deletions, for each of the n = 50 ancient genomes in the refined dataset (after applying additional ancestry state filters and removing outlier genomes). (TIF) [file pcbi.1010788.s015.tif]

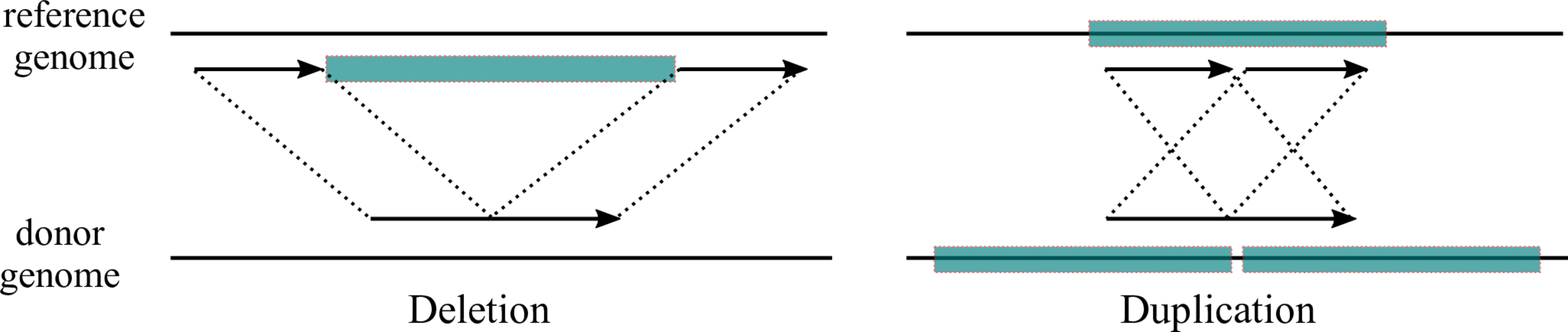

Supplement: S15 Fig — We use short-read Illumina mappings in a BAM file as input. We split each discordant read (whose mapping quality is larger than the given threshold and does not overlap with a known satellite) from the middle, keeping the initial mapping as one element and the other subsequence (split segment) as the second element of a pair. We remap the split segment to the reference genome, and evaluate the position and the orientation of both reads to identify the presence of putative CNVs. (TIF) [file pcbi.1010788.s016.tif]
