# Supplementary material for: CONGA: Copy number variation genotyping in ancient genomes and low-coverage sequencing data
Source: PLoS Comput Biol. 2022 Dec 14;18(12):e1010788. doi: 10.1371/journal.pcbi.1010788 (PMC9873172; doi:10.1371/journal.pcbi.1010788)
Supplement: Attachment — Submitted filename: Response_to_reviews.pdf [file pcbi.1010788.s021.pdf]

Dear Drs. Marc Robinson-Rechavi and Sushmita Roy,

We thank you and the reviewers for your helpful suggestions on our manuscript “CONGA: Copy number variation genotyping in ancient genomes and low-coverage sequencing data”. We revised the manuscript following these points.

Below we summarize the main changes to our work:

- Reviewers 1 and 3 had noted they were unable to run the algorithm. Apparently at least one case involved issues with the dependencies, and we now updated our Github README file accordingly to avoid similar complications.

- Addressing Reviewer 1 and 3's concerns about Table 1 being difficult to interpret, we added a new Figure (Figure 1), which describes the precision-recall curves of each algorithm together with the F-score comparison. We kept the previous Table 1 as Supplemental Table 1.A.

- Following Reviewer 3’s suggestion, we now describe the overlap between GenomeSTRiP- and CONGA-predicted events (as part of Supplemental Table 1).

- Following Reviewer 2’s suggestion, we added a new section discussing the treatment of overlapping CNVs.

- Based on Reviewer 3's suggestion, we moved the split-read section from the Methods section to Supplemental Notes S4, in order to improve the readability of the manuscript.

- We made stylistic modifications to Figure 3 (previously Figure 2) and Figure 4 (previously Figure 4) following suggestions by Reviewer 1 and Reviewer 3.

The updated sections are highlighted in red within the resubmitted manuscript document.

Please also find below our point-by-point response to the Reviewers.

We hope you and the reviewers find our responses and the revised version satisfactory.

Please let us know if you have any questions or comments.

Best wishes,

Arda Söylev, on behalf of all authors

CONGA Reviews

Reviewer 1

The authors present CONGA, a novel method to genotype individuals on a provided set of copy number variations (CNVs), that are marked as either deletions or duplications. Genotyping, rather than discovering CNVs, means that this method can be applied at lower sequencing depths, and can therefore be used to genotype CNVs in ancient genomes as well. The authors assess their method with the use of simulated ancient data, as well as real ancient data. Overall, the manuscript is well-written, follows a logical order, and is quite clear. Unfortunately, I was not able to compile and test CONGA, as I got compilation errors for one of the dependencies (sonic). It would be nice if the authors could add CONGA to bioconda, to make it easier for users to install the tool.

We thank the Reviewer for their constructive comments.

Regarding the compilation issue, we believe it would be solved by cloning the repository using the “--recursive” flag.

The overall command should be: “`git clone https://github.com/asylvz/CONGA --recursive`”, as described in the README file. We hope this solves the issue.

We agree that adding CONGA to bioconda would be a nice feature, however this would not be straightforward since our code was written in C. We noted this and will definitely consider it for the future.

1- Lines 147-148: Please mention in the main text that the pre-publication quality filtering only applies to the Yamnaya genome. The current phrasing makes it sound like all ancient genomes had this issue.

Now added, thank you for the suggestion.

2- Table 1: There is a lot of information condensed into this table, which makes it difficult to compare between methods easily.

Thank you for the helpful suggestion. We now moved the table to Supplemental Material and added Figure 1 (Figure R1 below) instead.

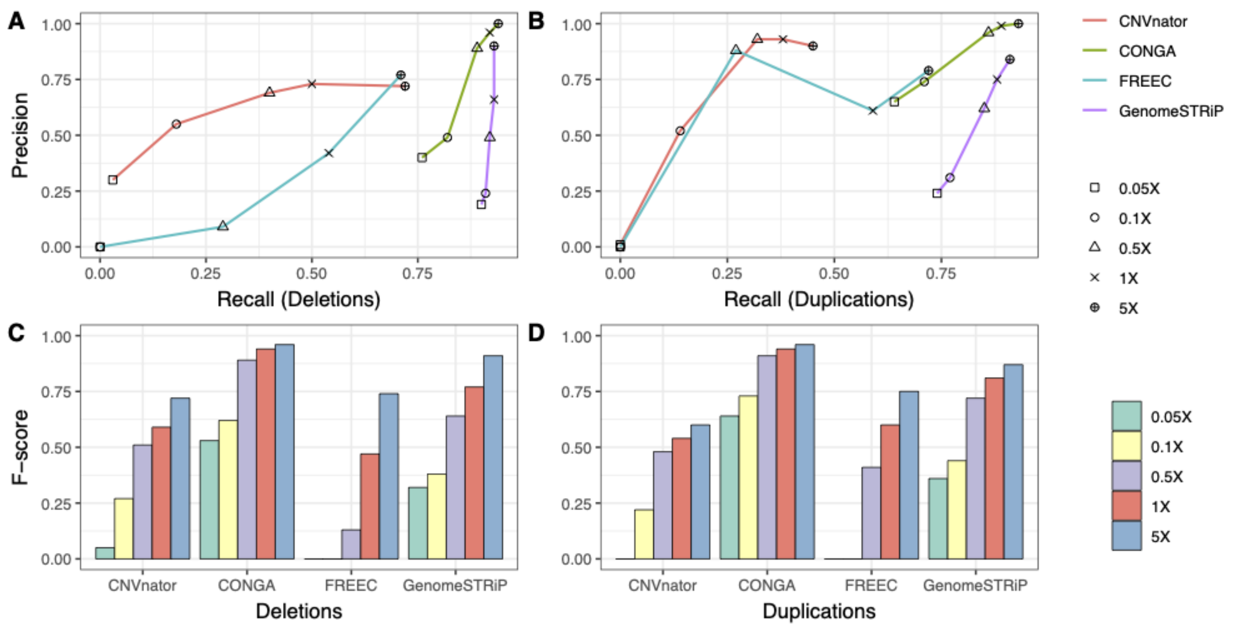

Figure R1: The figure shows CNV prediction performances of CONGA, GenomeSTRiP, FREEC and CNVnator on simulated genomes with depths 0.05x, 0.1x, 0.5x, 1x and 5x, for deletions and for duplications. In (A) and (B), we show recall-precision curves based on depths of coverage values for deletions and duplications, respectively. In (C) and (D), we show the F-scores, calculated as (2 x Precision x Recall) / (Precision + Recall). The figures represent the average statistics calculated for medium (1 kbps - 10 kbps) and large (10 kbps - 100 kbps) CNVs. See Supplemental Table S1A for detailed information including small (100 bps - 1 kbps) CNVs, as well as mrCaNaVaR predictions for large variations. Commands used to run each tool are provided in Supplemental Material. The results here were generated using the cutoff C-Score <0.5 for CONGA, while no read-pair or mappability filters were applied.

3- Figure 2: The y axis should be consistent between subplots. Ideally, the value of 1.0 should also be included in the y-axis as the theoretical maximum value.

We modified the figure accordingly (Figure R2 below).

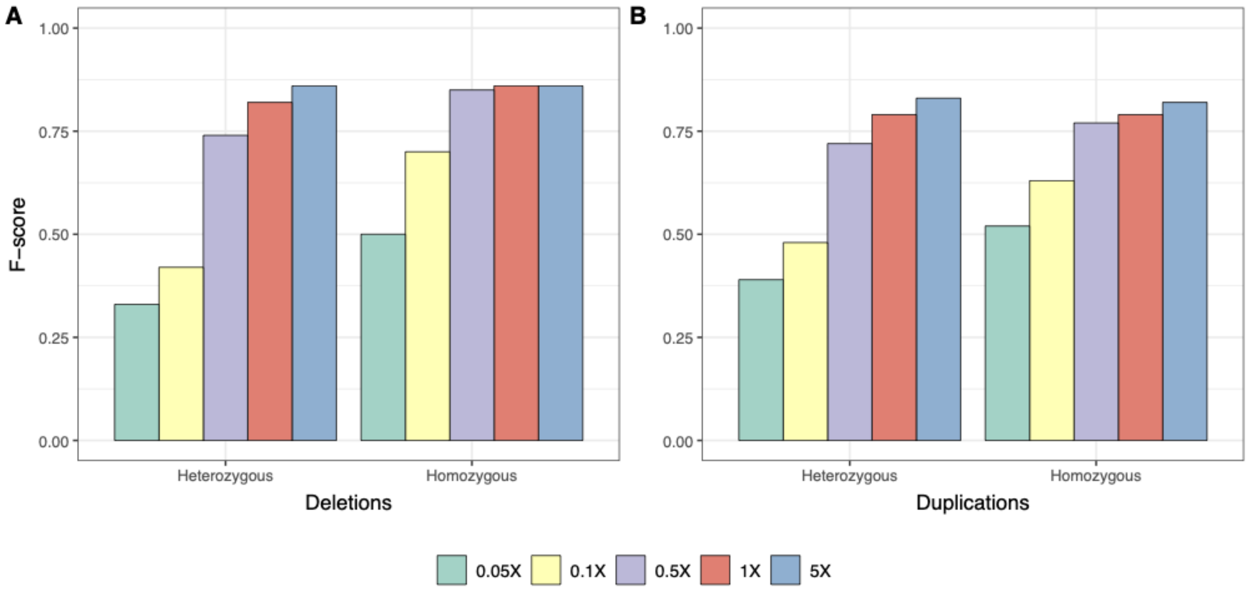

Figure R2: Performance (F-scores) of CONGA in correctly inferring copy-numbers of (A) deletions and (B) duplications using merged sets of medium and large CNVs, at various coverage values.

4- Figure 4: The colour scheme used makes some labels hard to read in print (especially the light blues in 4A (e.g. Kolyma_River).

We thank the Reviewer for this suggestion. We changed our color scheme and we hope the colors in the new version are more readily distinguishable (Figure R3 below).

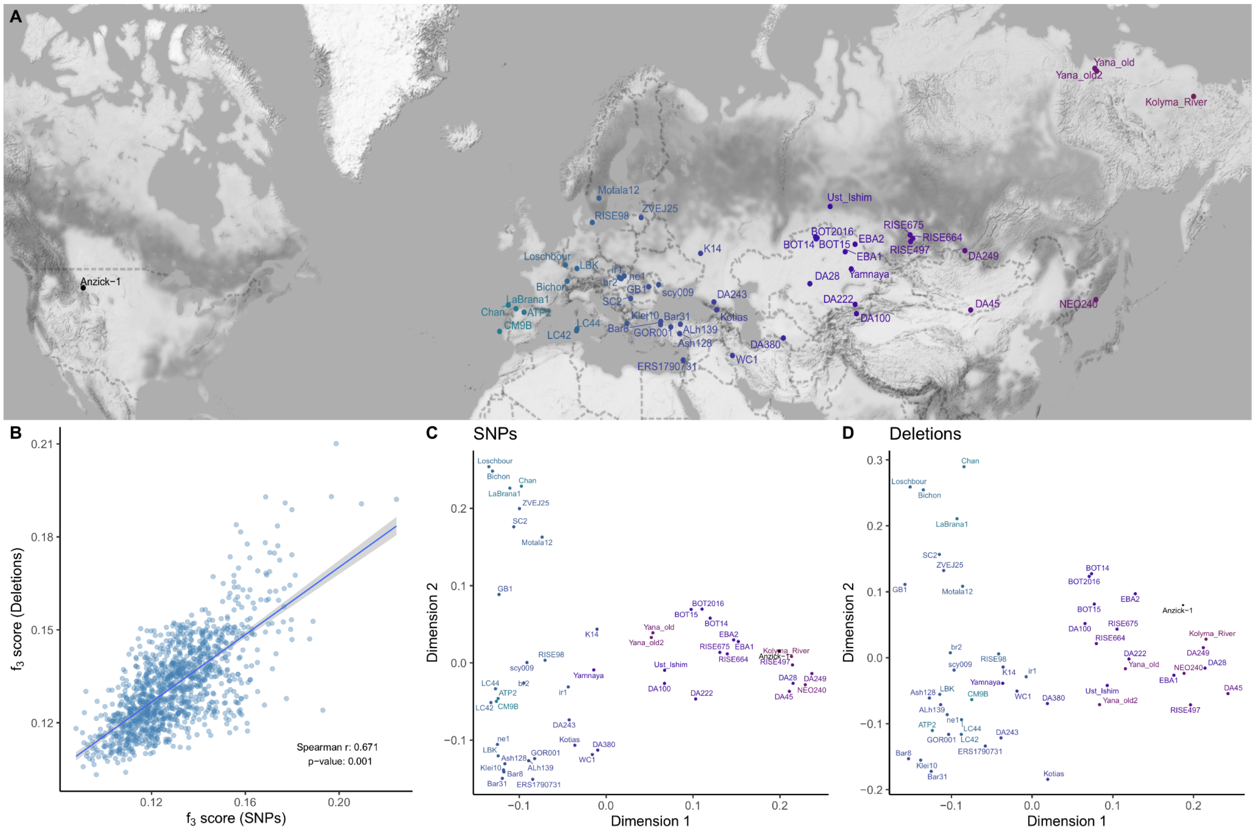

Figure R3: (A) Geographic locations of the 50 ancient individuals used in the analyses. (B) Comparison of genetic distances calculated using SNPs and deletions. We calculated the Spearman correlation coefficient between two matrices and then calculated the Mantel test p-value using the "mantel" function in R package "vegan" (v2.5-7). (C) and (D) represent multidimensional scaling plots that summarize outgroup-f3 statistics calculated across all pairs among the 56 ancient individuals using SNPs and deletions, respectively.

Reviewer 2

The authors propose CONGA, a specialized tool for the genotyping of copy-number variation in ancient genomes, which come with a plethora of challenges owing to low coverage, sample damage, and issues in library preparation. CONGA uses any given set of CNV calls as a truth set for genotyping using read-depth and split-read methodologies.

The authors evaluate the performance of their algorithm on simulated data, down-sampled real genomes, as well 71 ancient genomes, in a manner spanning a wide range of coverages and diverse ancestry.

The paper is written well - its question is clear, the goals of each section are appropriate, described effectively and concisely.

The authors do an excellent job of describing not only the strengths and applications of their algorithm, but also its faults, caveats, and areas of underperformance. The paper highlights the usability of CONGA while explaining clearly areas the tool is not suitable for analysis (such as genotyping ancient genomes in <5x coverage).

Additionally, the algorithm performs as expected. True-positive rate, as well as false-discovery rate largely scale with coverage. The algorithm tends to perform better for deletions than other types of CNVs, and CONGA is most performant for variants > 1kb as expected of short-read datasets.

The results are clear and the tool looks useful for those who are interested in genotyping CNVs in low-coverage ancient genomes. Overall, CONGA is deserving of publication in this journal without revision.

We thank the Reviewer for their encouraging comments.

1- Page 2, Line 83 - The CONGA algorithm is initially described in terms of read-depth and split-reads. However, a small line should be inserted here to quickly inform of the reader of how CONGA uses this information differently from already available read-depth and split-read callers.

Thank you. We now added the sentence in lines 81 - 83: *“We note that an alternative CNV genotyping tool, GenomeSTRiP (Handsaker et al., 2011; Handsaker et al., 2015), also uses similar information but is mainly designed for genotyping multiple genomes simultaneously, and evaluates the read depth data using Gaussian mixture models instead of Poisson.”*

2- Page 3, Line 119 - The authors title this section 'copy number predictions of CNVs' but note that CONGA does not evaluate >= 3 copies. This section should be renamed, as many working

on CNVs may assume more capability here as opposed to simply detecting homozygous vs homozygous variation.

This is indeed a good point; we changed the title as: "*Diploid genotype inference*"

3- The authors mention that their input callset was determined across 4 datasets by choosing non-overlapping variants, but some polymorphic CNVs in the population may have overlapping breakpoints and thus such a heuristic may filter out genotype-able CNVs. It would be worth attempting to decide on a way to keep some of these CNVs in the input callset, perhaps based on some metric of reciprocal overlap.

We agree with the Reviewer on this limitation. We also noticed that we had not explained the reason for excluding overlapping CNVs in sufficient detail in the previous version of the manuscript. There are two issues. First, CNV breakpoint resolution of CNV callers can frequently be imprecise, such that overlapping CNVs might actually be representing the same event called with slightly different breakpoints (e.g. with 100 bp distance) on different genomes or with different tools. Second, having overlapping CNVs in the input set (i.e. the call set) creates the risk of calling multiple events although only one event is actually present. For instance, if a small deletion resides within a larger deletion, a genome carrying the larger deletion in heterozygous state would be automatically genotyped heterozygous for the smaller deletion. We thus preferred to be conservative and omitted overlapping CNVs. Specifically, if two CNVs overlaped >50% of their size, we excluded the smaller one.

This creates a trade-off in that we do not analyse some events. Also, our calls could still be affected by real overlapping events not in our input list. We hence agree with the Reviewer that developing a strategy to accurately genotype overlapping events could be possible (e.g. based on a serial evaluation of likelihoods of overlapping events). However, developing such an algorithm is non-trivial and would be a study on its own.

We mention these points now in the manuscript in lines 459 - 470.

Reviewer 3

The authors present CONGA, a software to detect copy number variants (CNVs) in low-coverage genomes, e.g., ancient genomes based on a list of CNVs. he authors benchmark the tool using simulations and real ancient genomes down sampled to different depths.
CNVs are of great interest in evolutionary genomics as they are often subject to selection. The discovery of new CNVs is already challenging with modern high-coverage genomes. Thus, it is understandable that the authors focus on the detection of given CNVs in low-coverage genomes. CONGA will be an asset for the analyses of ancient genomes.
The manuscript is nicely written and easy to read. I appreciate the details the authors provide for all analyses, especially also the exact commands they were using listed in the supplement information.

We thank the Reviewer for their encouraging comments.

General comment:
My main concern is about the paired-read approach to detect duplications. I am not really sure that this approach is applicable in reality. The idea is to split the read to two equally sized subreads. My concern is that the split in the middle is only ideal, if the CNV breakpoint is at the same place. If the breakpoint is further away (what will be normally the case), one of the subreads will contain two parts of a sequence, making it difficult (or impossible) to map it to a correct location. In the discussion the authors mention that the paired-read approach is quite limited. I don't know if it would make the manuscript more appealing if this feature would be omitted in the manuscript and moved entirely to the supplement.

Thank you. We agree with the Reviewer's criticism, and thus decided to move the section on split-read analysis to Supplemental Note S4 in order to increase the readability of the paper.

Unfortunately, I was not able to test CONGA, as I was not able to compile the code. The library 'bzlib.h' seems to be missed (see also below).

The authors did a lot of great analyses, but their figures should be improved to adequately represent the work (see below).

These points we addressed below.

1- Table 1: I have a hard time to digest the table (too many numbers). It is difficult to see the trends. Would it be possible to make a figure out of the TPR, FGDR and F1 and add the table to the supplement?

We thank the Reviewer for this suggestion. We now moved the table to the Supplemental as, Table S1.A, and present the information in the new Figure 2 instead (see Figure R1 below).

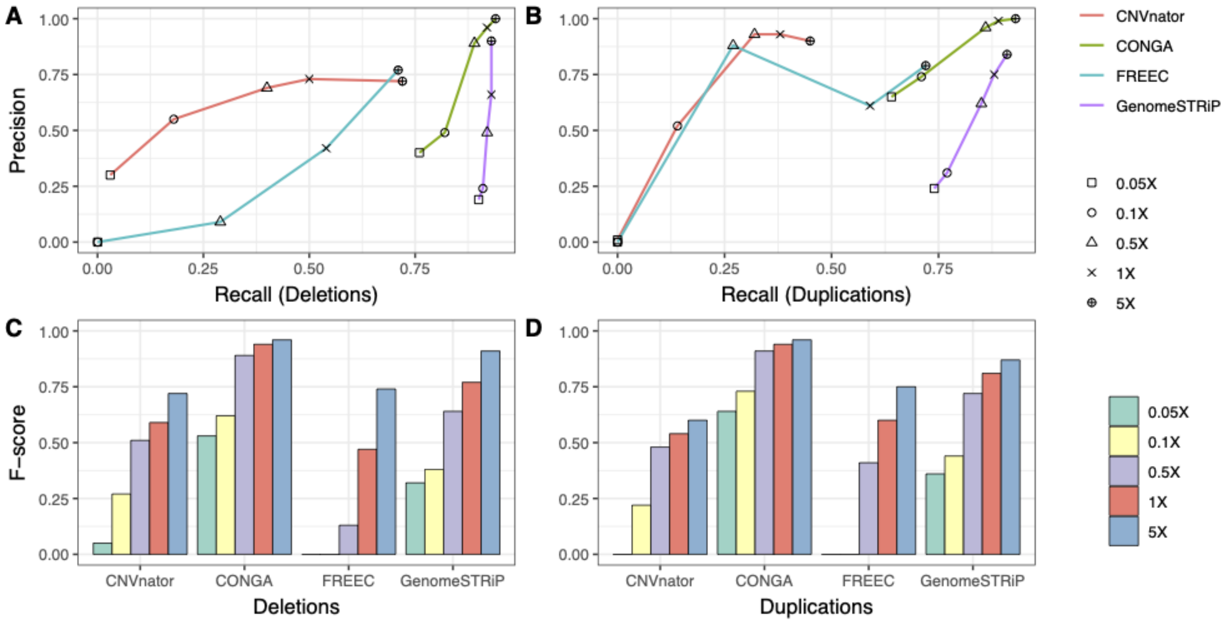

Figure R1: The figure shows CNV prediction performances of CONGA, GenomeSTRiP, FREEC and CNVnator on simulated genomes with depths 0.05x, 0.1x, 0.5x, 1x and 5x, for deletions and for duplications. In (A) and (B), we show recall-precision curves based on depths of coverage values for deletions and duplications, respectively. In (C) and (D), we show the F-scores, calculated as (2 x Precision x Recall) / (Precision + Recall). The figures represent the average statistics calculated for medium (1 kbps - 10 kbps) and large (10 kbps - 100 kbps) CNVs. See Supplemental Table S1A for detailed information including small (100 bps - 1 kbps) CNVs, as well as mrCaNaVaR predictions for large variations. Commands used to run each tool are provided in Supplemental Material. The results here were generated using the cutoff C-Score <0.5 for CONGA, while no read-pair or mappability filters were applied.

2- Figure 2: Please use the same scale for deletions and duplications. Maybe it would even be possible to merge the two graphs having as x-axis the different coverage. This would allow to compare the deletions and duplications more easily.

Thank you. We modified the figure accordingly, fixing the axes to [0-1] (see Figure R2 below). We also considered the Reviewer's suggestion, but our main aim here was to compare CONGA's performance for homozygous and heterozygous events (not comparing deletions and duplications). We therefore thought that the current version would be more easily interpretable than one that joins information on deletions and duplications together.

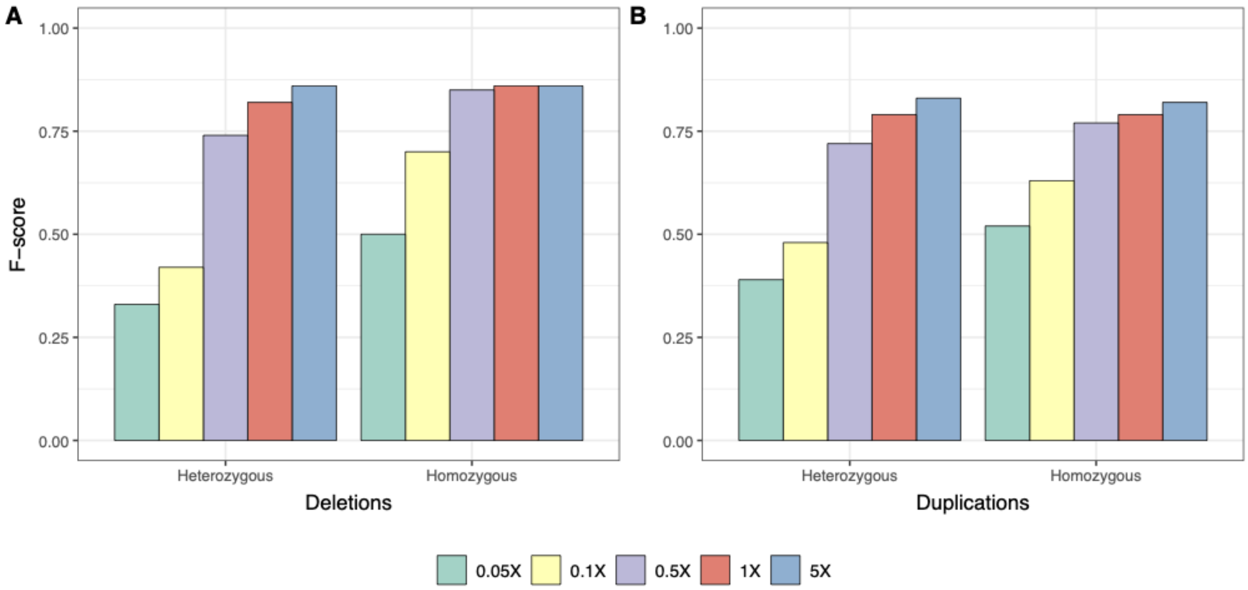

Figure R2: Performance (F-scores) of CONGA in correctly inferring copy-numbers of (A) deletions and (B) duplications using merged sets of medium and large CNVs, at various coverage values.

3- Figure 3: Here, I think you want to show the performance of CONGA at different depths. If this is the case, the x-axis should show the depths. The current figure is difficult to read/understand.

We thank the Reviewer for the comment and suggestion. Our main aim in this figure is to show both (1) how the performance of CONGA is affected from the coverage variations in real genomes, but as importantly, (2) to show how CONGA's duplication calling accuracy in the Yamnaya genome is much lower than the other two genomes (presumably due to pre-publication filtering, as we discuss in Supplemental Note S2). We did create a plot following Reviewer's suggestion (see Figure R4 below), but we felt that using the current TPR-FDR plot was more helpful in making the second point. We believe the Reviewer agrees with this, but we are open to any other suggestions.

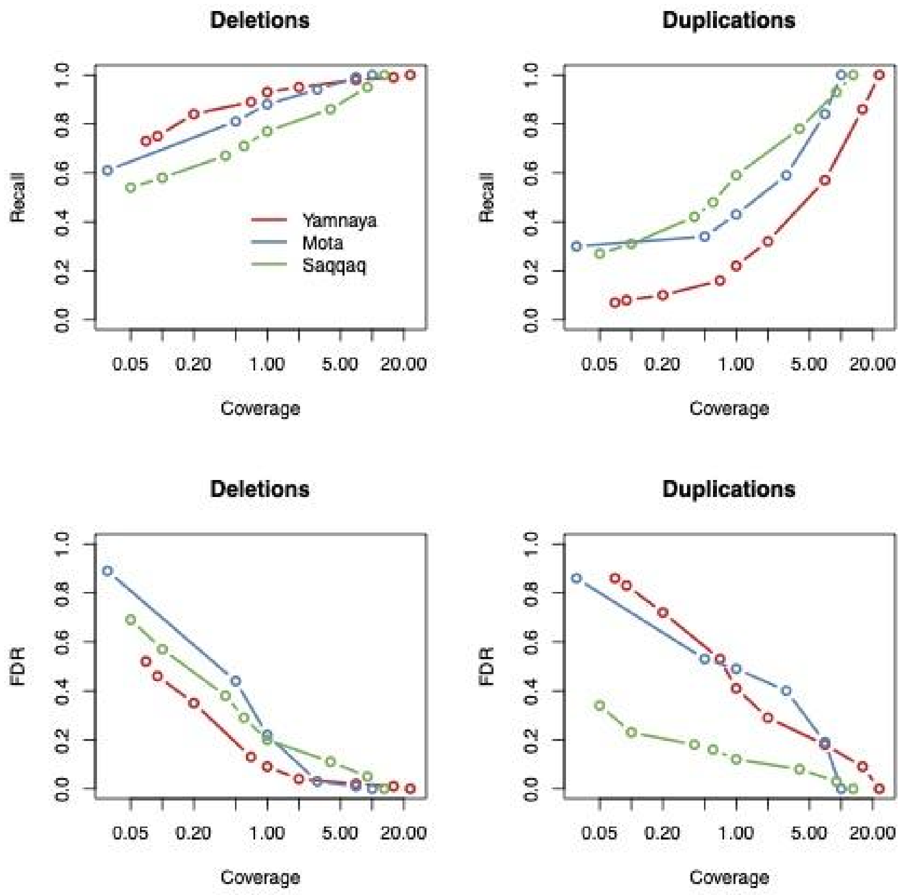

Figure R4: Recall (TPR) vs FDR curves for deletion and duplication predictions of CONGA using Mota, Saqqaq and Yamnaya genomes down-sampled to various depths from their original coverages of 9.6x, 13.1x and 23.3x, respectively. The x-axis shows the down-sampled coverage values. We calculated recall (TPR) and FDR for down-sampled genomes assuming that our CONGA-based predictions with the original genomes (full data) reflect the ground truth. These predictions, in turn, were made using modern-day CNVs as candidate CNV list. The purpose of the experiment was to evaluate accuracy at lower coverage relative to the full data, as well as to compare performance across different real genomes (Methods)

4- Figure 4:

- *Please change the colors. The current ones are difficult to distinguish.*
- *B+C, is there any reason why dimension 2 is on the x-axis? If not, I would put dimension 1 on the x-axis as it is normally done.*
- *Adding directly, “SNP” and “CNV” to plot B and C, respectively, would help to read them.*

We thank the Reviewer for these suggestions. We changed our color scheme and added titles to panels B and C as “SNPs” and “Deletions”, respectively. In the previous version, in panels B

and C, we had actually shown dimension 1 on the x-axis, but plotted the wrong labels accidentally. We now corrected the axis labels, and thank the Reviewer for their attention. See Figure R5 below.

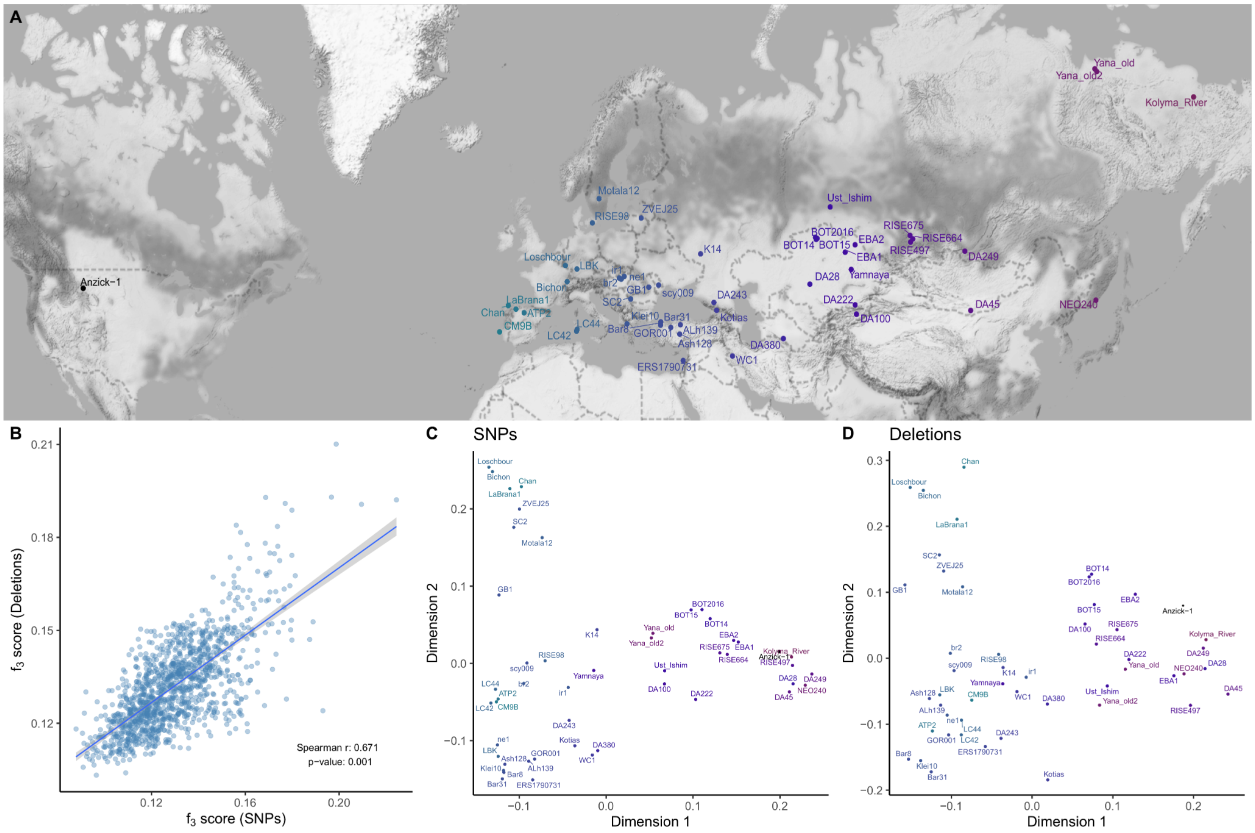

Figure R5: (A) Geographic locations of the 50 ancient individuals used in the analyses. (B) Comparison of genetic distances calculated using SNPs and deletions. We calculated the Spearman correlation coefficient between two matrices and then calculated the Mantel test p-value using the "mantel" function in R package "vegan" (v2.5-7). (C) and (D) represent multidimensional scaling plots that summarize outgroup-f3 statistics calculated across all pairs among the 56 ancient individuals using SNPs and deletions, respectively.

5- Line 573: Why is here the seeding for the mapping not removed (param -l)?

We thank the Reviewer for noticing this omission. We had indeed used the "–l 16500" parameter to disable seed but had missed mentioning this in the methods. This information is now also added in line 502.

6- Line 618: If the SNPs were bi-allelic, then I don't see how the minor allele frequency could be zero? Omit the text in parenthesis?

Thank you for pointing this out. The SNPs were chosen to be bi-allelic in modern-day African samples, but they can still have 0 MAF in our ancient Eurasian sample. We revised the text to clarify this point and we hope the current version avoids such confusion (lines 605-609): *"Following the same reasoning as above regarding ascertainment bias, we used the African population as an ascertainment population to create an SNP genotyping set for calling SNPs in the ancient genomes. To create this dataset, we started with all bi-allelic SNPs in the 1000 Genomes Project phase 3 dataset (The 1000 Genomes596 Project Consortium, 2015) and selected the SNPs with a minor allele frequency greater than zero in 661 African genomes of the 1000 Genomes Project Phase 3."*

7- Line 632: Change 'represent' to 'present'

Thank you, but we actually did mean "represent". The sentence intended to explain that deletions not fully mapping to chimp or bonobo genomes most probably represent (i.e. stand for) derived insertions in the human lineage, and we had thus removed them from our deletion list. We hope this was clear.

8- Line 202: So finally, the remaining samples are 'only' WSG samples (no captured ones)?

Thank you for the suggestion. We changed this part as *"Consequently we removed these 21 genomes from further analyses, thus retaining only shotgun-sequenced genomes with coverage >0.4x."*

9- Line 235: Any reference or rationales for this hypothesis? If not, you cannot test one-sided (line242). I am not sure that you can call this "slightly lower" when the one-sided test results in a p-value of 0.055. At the best this is a tendency.

Thank you for this point.

We changed the first sentence to: *"If deletions are under negative selection we may also expect longer deletions, or deletions containing evolutionary conserved genes, to be segregating at lower frequencies, \AS{similar to that observed with modern-day human genomes (Conrad et al., 2010; Cooper et al., 2011; Sudmant et al., 2015)."*

We also changed the *"slightly lower dN/dS values"* to *"showed a tendency to disrupt genes with lower dN/dS values".* We hope these changes address the Reviewer's criticism.

10- Line258: Would be good to get the computer specs already here (and not to have to wait until line 266). So the applications were run multithreaded with 16 cores?

Thank you. We agree with the suggestion and moved the computer specs part to the beginning of the paragraph (line 267). We also added a line explaining that we ran the algorithm single threaded.

11- Line 294: Do CONGA and GenomeSTRiP find the same deletions? What is the overlap?

Thank you for this question. We added the following line to the main text based on the Table below (also added this data to the Supplemental Table 1A)

"We also found that 96% of deletion and 89% of duplication predictions of CONGA overlap with those of GenomeSTRiP (calculated as the average match rate of medium and large sized CNVs). The predictions by GenomeSTRiP match those of CONGA at lower rates (c.55%), mainly owing to higher FDR in GenomeSTRiP results (also see Supplemental Table S1.A for the comparison between CONGA and GenomeSTRiP)."

Table R1: The table shows a comparison between CONGA and GenomeSTRiP predictions for various depths of coverage.

	**Large CNVs**					
	**Deletions**			**Duplications**		
	**Match**	**Total CONGA**	**Total GSTRiP**	**Match**	**Total CONGA**	**Total GSTRiP**
**0.05X**	2954	3020	7221	1580	1824	5119
**0.1X**	2495	2559	5708	1490	1639	4133
**0.5X**	1435	1450	2831	1375	1477	2341
**1X**	1339	1352	2097	1446	1369	2021
**5X**	1298	1303	1566	1368	1437	1861
**Average match rate**	-	**0.986**	**0.564**	-	**0.943**	**0.541**
	**Medium CNVs**					
	**Deletions**			**Duplications**		
	**Match**	**Total CONGA**	**Total GSTRiP**	**Match**	**Total CONGA**	**Total GSTRiP**
**0.05X**	2719	2838	7091	1079	1327	4859
**0.1X**	2439	2581	5828	1100	1358	3798
**0.5X**	1489	1593	2954	1198	1395	1931
**1X**	1440	1557	2192	1277	1562	1653
**5X**	1461	1577	1604	1371	1554	1501
**Average match rate**	-	**0.938**	**0.575**	-	**0.836**	**0.564**

Overall (medium, large) average CONGA deletion match rate	**0.962**	*Overall (medium, large) average CONGA duplication match rate*	**0.890**
Overall (medium, large) average GenomeStrip deletion match rate	**0.569**	*Overall (medium, large) average GenomeStrip duplication match rate*	**0.553**

12- Github:

- *In README: Downloading and running: the step to go into the CONGA folder (cd CONGA) is missing.*
- *Not able to compile the code, and thus not able to test the software. I get the following error:*

```
$ make libs
make -C htslib
make[1]: Entering directory '/work/***redacted to protect reviewer anonymity***/CONGA/htslib'
gcc -g -Wall -O2 -I.  -c -o cram/cram_io.o cram/cram_io.c
cram/cram_io.c:52:10: fatal error: bzlib.h: No such file or directory
   52 | #include <bzlib.h>
      |          ^~~~~~~~~
compilation terminated.
make[1]: *** [Makefile:132: cram/cram_io.o] Error 1
make[1]: Leaving directory '/work/***redacted to protect reviewer anonymity***/CONGA/htslib'
make: *** [Makefile:25: libs] Error 2
```

We thank the Reviewer for this point. We now included the “cd CONGA” command in the README file.

We believe that the compilation issue the Reviewer ran into is related to a requirement of the “htslib” library. This can be solved by simply running the following command: **`“sudo apt-get install libbz2-dev”`**. We also added this information to our README on Github.
