# Supplementary material for: CONGA: Copy number variation genotyping in ancient genomes and low-coverage sequencing data
Source: PLoS Comput Biol. 2022 Dec 14;18(12):e1010788. doi: 10.1371/journal.pcbi.1010788 (PMC9873172; doi:10.1371/journal.pcbi.1010788)
Supplement: Attachment — Submitted filename: Response_editor_AS.pdf [file pcbi.1010788.s022.pdf]

Dear Drs. Marc Robinson-Rechavi and Sushmita Roy,

We thank you and the reviewers for the suggestions on our manuscript “CONGA: Copy number variation genotyping in ancient genomes and low-coverage sequencing data”.

The issue that you and the reviewer experienced about compiling CONGA is caused by one of the libraries that we use in our algorithm, HTSlib. This library is one of the most widely used C libraries for reading/writing high-throughput sequencing data. Although installing one of the libraries that HTSlib uses (libcurl) will solve the problem that you encountered, we added other alternatives for the users of our program because we believe that our algorithm will be used by many studies in the future and we value reproducibility.

Below are our updates:

- We updated our readme file in our github page (https://github.com/asylvz/CONGA/) and added Linux commands that one needs in order to download all the required development libraries.
- We added an option to compile our code for people with no sudo access. However some options will be disabled and “libz” library is still needed.
- We uploaded a linux binary file under release v1.0 so that linux users (with X86_64 machine) can directly run CONGA without any compilation.
- We also added a Docker image, which is another alternative to run CONGA using a virtual machine.

We also asked multiple colleagues to test installation and we received positive feedback.

We hope you find the revised version satisfactory. Please let us know if you still encounter any issues or you have any questions or comments.

Best wishes,

Arda Söylev, on behalf of all authors
