# Supplementary material for: CONGA: Copy number variation genotyping in ancient genomes and low-coverage sequencing data
Source: PLoS Comput Biol. 2022 Dec 14;18(12):e1010788. doi: 10.1371/journal.pcbi.1010788 (PMC9873172; doi:10.1371/journal.pcbi.1010788)
Supplement: Attachment — Submitted filename: Review.docx [file pcbi.1010788.s019.docx]

The authors present CONGA, a software to detect copy number variants (CNVs) in low-coverage genomes, e.g., ancient genomes based on a list of CNVs. he authors benchmark the tool using simulations and real ancient genomes down sampled to different depths.

CNVs are of great interest in evolutionary genomics as they are often subject to selection. The discovery of new CNVs is already challenging with modern high-coverage genomes. Thus, it is understandable that the authors focus on the detection of given CNVs in low-coverage genomes. CONGA will be an asset for the analyses of ancient genomes.

The manuscript is nicely written and easy to read. I appreciate the details the authors provide for all analyses, especially also the exact commands they were using listed in the supplement information.

General comment:

My main concern is about the paired-read approach to detect duplications. I am not really sure that this approach is applicable in reality. The idea is to split the read to two equally sized subreads. My concern is that the split in the middle is only ideal, if the CNV breakpoint is at the same place. If the breakpoint is further away (what will be normally the case), one of the subreads will contain two parts of a sequence, making it difficult (or impossible) to map it to a correct location. In the discussion the authors mention that the paired-read approach is quite limited. I don’t know if it would make the manuscript more appealing if this feature would be omitted in the manuscript and moved entirely to the supplement.

Unfortunately, I was not able to test CONGA, as I was not able to compile the code. The library ‘bzlib.h’ seems to be missed (see also below).

The authors did a lot of great analyses, but their figures should be improved to adequately represent the work (see below).

Specific comments

Table 1: I have a hard time to digest the table (too many numbers). It is difficult to see the trends. Would it be possible le to make a figure out of the TPR, FGDR and F1 and add the table to the supplement?

Figure 2: Please use the same scale for deletions and duplications. Maybe it would even be possible to merge the two graphs having as x-axis the different coverage. This would allow to compare the deletions and duplications more easily.

Figure 3: Here, I think you want to show the performance of CONGA at different depths. If this is the case, the x-axis should show the depths. The current figure is difficult to read/understand.

Figure 4:

- Please change the colors. The current ones are difficult to distinguish.
- B+C, is there any reason why dimension 2 is on the x-axis? If not, I would put dimension 1 on the x-axis as it is normally done.
- Adding directly, “SNP” and “CNV” to plot B and C, respectively, would help to read them.

Line 573: Why is here the seeding for the mapping not removed (param -l)?

Line 618: If the SNPs were bi-allelic, then I don’t see how the minor allele frequency could be zero? Omit the text in parenthesis?

Line 632: Change ‘represent’ to ‘present’

Line 202: So finally, the remaining samples are ‘only’ WSG samples (no captured ones)?

Line 235: Any reference or rationales for this hypothesis? If not, you cannot test one-sided (line242). I am not sure that you can call this “slightly lower” when the one-sided test results in a p-value of 0.055. At the best this is a tendency.

Line258: Would be good to get the computer specs already here (and not to have to wait until line 266). So the applications were run multithreaded with 16 cores?

Line 294: Do CONGA and GenomeSTRiP find the same deletions? What is the overlap?

Github:

- In README: Downloading and running: the step to go into the CONGA folder (cd CONGA) is missing.
- Not able to compile the code, and thus not able to test the software. I get the following error:

$ make libs

make -C htslib

make[1]: Entering directory '/work/*****redacted to protect reviewer anonymity*****/CONGA/htslib'

gcc -g -Wall -O2 -I. -c -o cram/cram_io.o cram/cram_io.c

cram/cram_io.c:52:10: fatal error: bzlib.h: No such file or directory

52 | #include <bzlib.h>

| ^~~~~~~~~

compilation terminated.

make[1]: *** [Makefile:132: cram/cram_io.o] Error 1

make[1]: Leaving directory '/work/*****redacted to protect reviewer anonymity*****/CONGA/htslib'

make: *** [Makefile:25: libs] Error 2
