# Supplementary material for: CONGA: Copy number variation genotyping in ancient genomes and low-coverage sequencing data
Source: PLoS Comput Biol. 2022 Dec 14;18(12):e1010788. doi: 10.1371/journal.pcbi.1010788 (PMC9873172; doi:10.1371/journal.pcbi.1010788)
Supplement: Attachment — Submitted filename: CONGA_review1.docx [file pcbi.1010788.s020.docx]

The authors present CONGA, a novel method to genotype individuals on a provided set of copy number variations (CNVs), that are marked as either deletions or duplications. Genotyping, rather than discovering CNVs, means that this method can be applied at lower sequencing depths, and can therefore be used to genotype CNVs in ancient genomes as well. The authors assess their method with the use of simulated ancient data, as well as real ancient data.

Overall, the manuscript is well-written, follows a logical order, and is quite clear. Unfortunately, I was not able to compile and test CONGA, as I got compilation errors for one of the dependencies (sonic). It would be nice if the authors could add CONGA to bioconda, to make it easier for users to install the tool.

Specific comments:

- Lines 147-148: Please mention in the main text that the pre-publication quality filtering only applies to the Yamnaya genome. The current phrasing makes it sound like all ancient genomes had this issue.
- Table 1: There is a lot of information condensed into this table, which makes it difficult to compare between methods easily.
- Figure 2: The y axis should be consistent between subplots. Ideally, the value of 1.0 should also be included in the y-axis as the theoretical maximum value.
- Figure 4: The colour scheme used makes some labels hard to read in print (especially the light blues in 4A (e.g. Kolyma_River).
